# Supplementary material for: A Humanized Yeast Phenomic Model of Deoxycytidine Kinase to Predict Genetic Buffering of Nucleoside Analog Cytotoxicity
Source: Genes (Basel). 2019 Sep 30;10(10):770. doi: 10.3390/genes10100770 (PMC6826991; doi:10.3390/genes10100770)
Supplement: Supplementary file 1 [file genes-10-00770-s001.zip › Additional_Files/Additional_File7_GOTermHeatmaps.zip!/Additional_File7_GOTermHeatmaps/tRNA_metabolic_process.pdf]

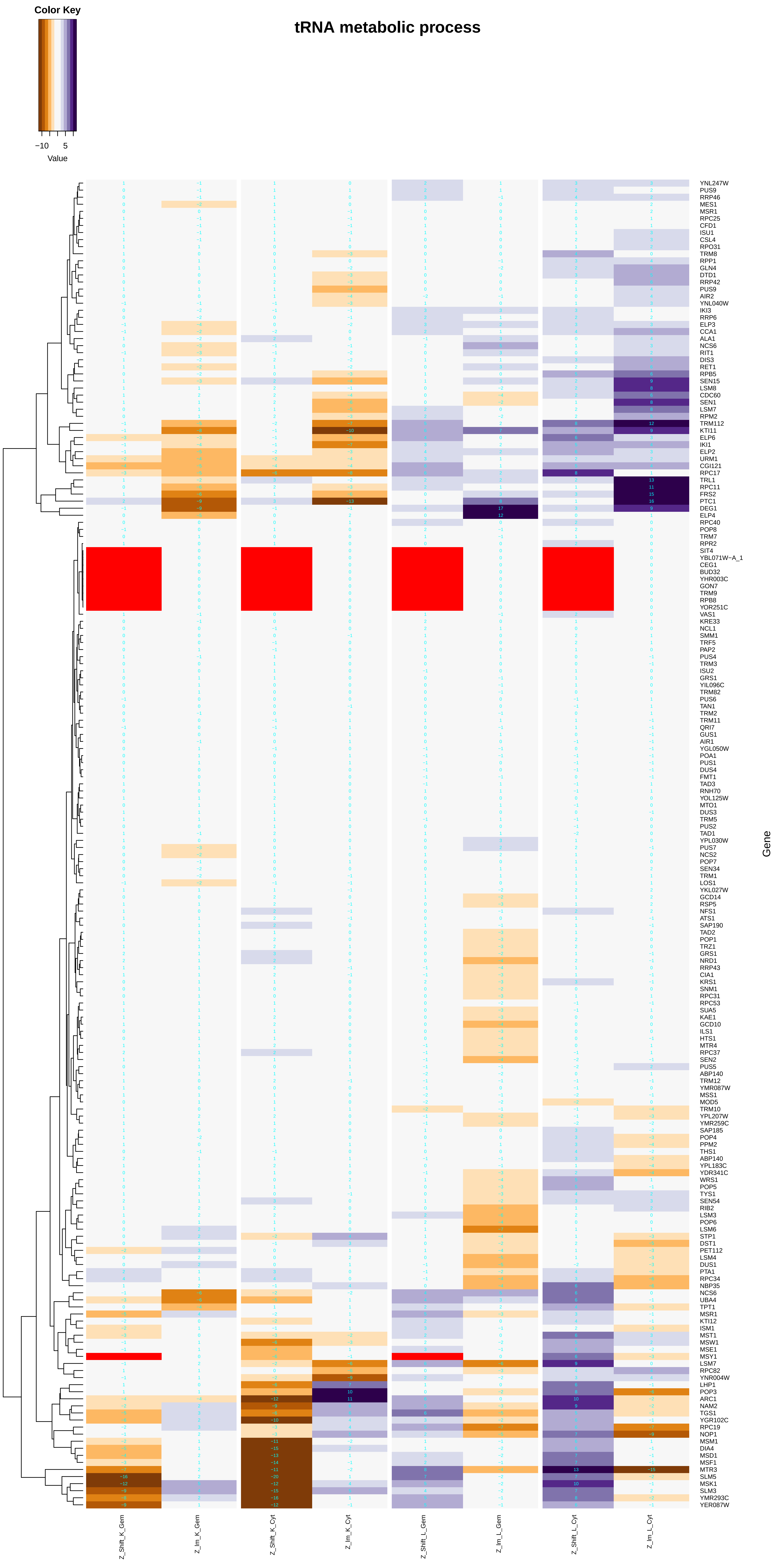
tRNA metabolic process
Color Key
−10
5
Value
Gene
Z_Shift_K_Gem
Z_Im_K_Gem
Z_Shift_K_Cyt
Z_Im_K_Cyt
Z_Shift_L_Gem
Z_Im_L_Gem
Z_Shift_L_Cyt
Z_Im_L_Cyt
YNL247W
PUS9
RRP46
MES1
MSR1
RPC25
CFD1
ISU1
CSL4
RPO31
TRM8
RPP1
GLN4
DTD1
RRP42
PUS9
AIR2
YNL040W
IKI3
RRP6
ELP3
CCA1
ALA1
NCS6
RIT1
DIS3
RET1
RPB5
SEN15
LSM8
CDC60
SEN1
LSM7
RPM2
TRM112
KTI11
ELP6
IKI1
ELP2
URM1
CGI121
RPC17
TRL1
RPC11
FRS2
PTC1
DEG1
ELP4
RPC40
POP8
TRM7
RPR2
SIT4
YBL071W−A_1
CEG1
BUD32
YHR003C
GON7
TRM9
RPB8
YOR251C
VAS1
KRE33
NCL1
SMM1
TRF5
PAP2
PUS4
TRM3
ISU2
GRS1
YIL096C
TRM82
PUS6
TAN1
TRM2
TRM11
QRI7
GUS1
AIR1
YGL050W
POA1
PUS1
DUS4
FMT1
TAD3
RNH70
YOL125W
MTO1
DUS3
TRM5
PUS2
TAD1
YPL030W
PUS7
NCS2
POP7
SEN34
TRM1
LOS1
YKL027W
GCD14
RSP5
NFS1
ATS1
SAP190
TAD2
POP1
TRZ1
GRS1
NRD1
RRP43
CIA1
KRS1
SNM1
RPC31
RPC53
SUA5
KAE1
GCD10
ILS1
HTS1
MTR4
RPC37
SEN2
PUS5
ABP140
TRM12
YMR087W
MSS1
MOD5
TRM10
YPL207W
YMR259C
SAP185
POP4
PPM2
THS1
ABP140
YPL183C
YDR341C
WRS1
POP5
TYS1
SEN54
RIB2
LSM3
POP6
LSM6
STP1
DST1
PET112
LSM4
DUS1
PTA1
RPC34
NBP35
NCS6
UBA4
TPT1
MSR1
KTI12
ISM1
MST1
MSW1
MSE1
MSY1
LSM7
RPC82
YNR004W
LHP1
POP3
ARC1
NAM2
TGS1
YGR102C
RPC19
NOP1
MSM1
DIA4
MSD1
MSF1
MTR3
SLM5
MSK1
SLM3
YMR293C
YER087W

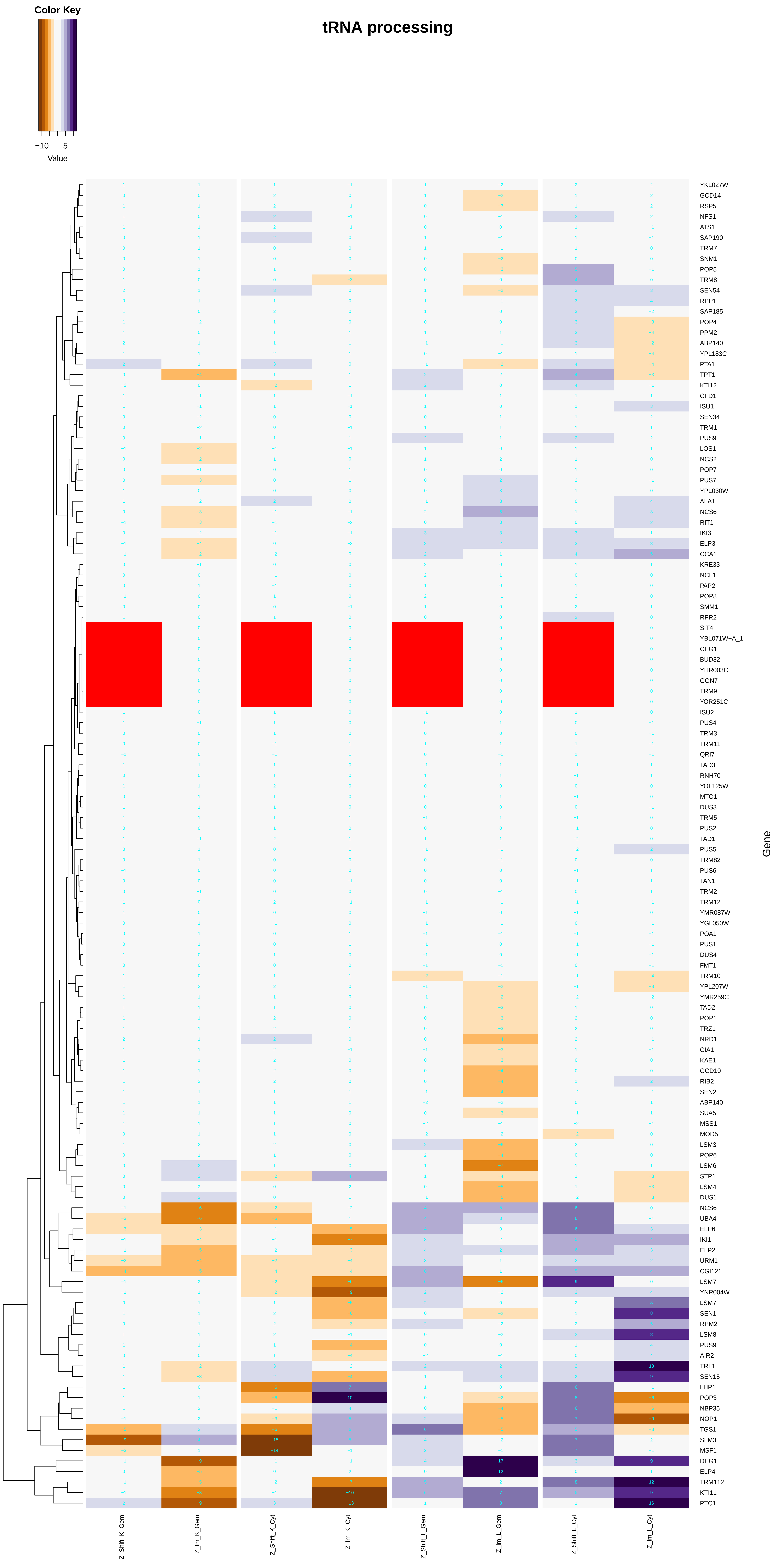
tRNA processing
Color Key
−10
5
Value
Gene
YKL027W
GCD14
RSP5
NFS1
ATS1
SAP190
TRM7
SNM1
POP5
TRM8
SEN54
RPP1
SAP185
POP4
PPM2
ABP140
YPL183C
PTA1
TPT1
KTI12
CFD1
ISU1
SEN34
TRM1
PUS9
LOS1
NCS2
POP7
PUS7
YPL030W
ALA1
NCS6
RIT1
IKI3
ELP3
CCA1
KRE33
NCL1
PAP2
POP8
SMM1
RPR2
SIT4
YBL071W−A_1
CEG1
BUD32
YHR003C
GON7
TRM9
YOR251C
ISU2
PUS4
TRM3
TRM11
QRI7
TAD3
RNH70
YOL125W
MTO1
DUS3
TRM5
PUS2
TAD1
PUS5
TRM82
PUS6
TAN1
TRM2
TRM12
YMR087W
YGL050W
POA1
PUS1
DUS4
FMT1
TRM10
YPL207W
YMR259C
TAD2
POP1
TRZ1
NRD1
CIA1
KAE1
GCD10
RIB2
SEN2
ABP140
SUA5
MSS1
MOD5
LSM3
POP6
LSM6
STP1
LSM4
DUS1
NCS6
UBA4
ELP6
IKI1
ELP2
URM1
CGI121
LSM7
YNR004W
LSM7
SEN1
RPM2
LSM8
PUS9
AIR2
TRL1
SEN15
LHP1
POP3
NBP35
NOP1
TGS1
SLM3
MSF1
DEG1
ELP4
TRM112
KTI11
PTC1
Z_Shift_K_Gem
Z_lm_K_Gem
Z_Shift_K_Cyt
Z_lm_K_Cyt
Z_Shift_L_Gem
Z_lm_L_Gem
Z_Shift_L_Cyt
Z_lm_L_Cyt

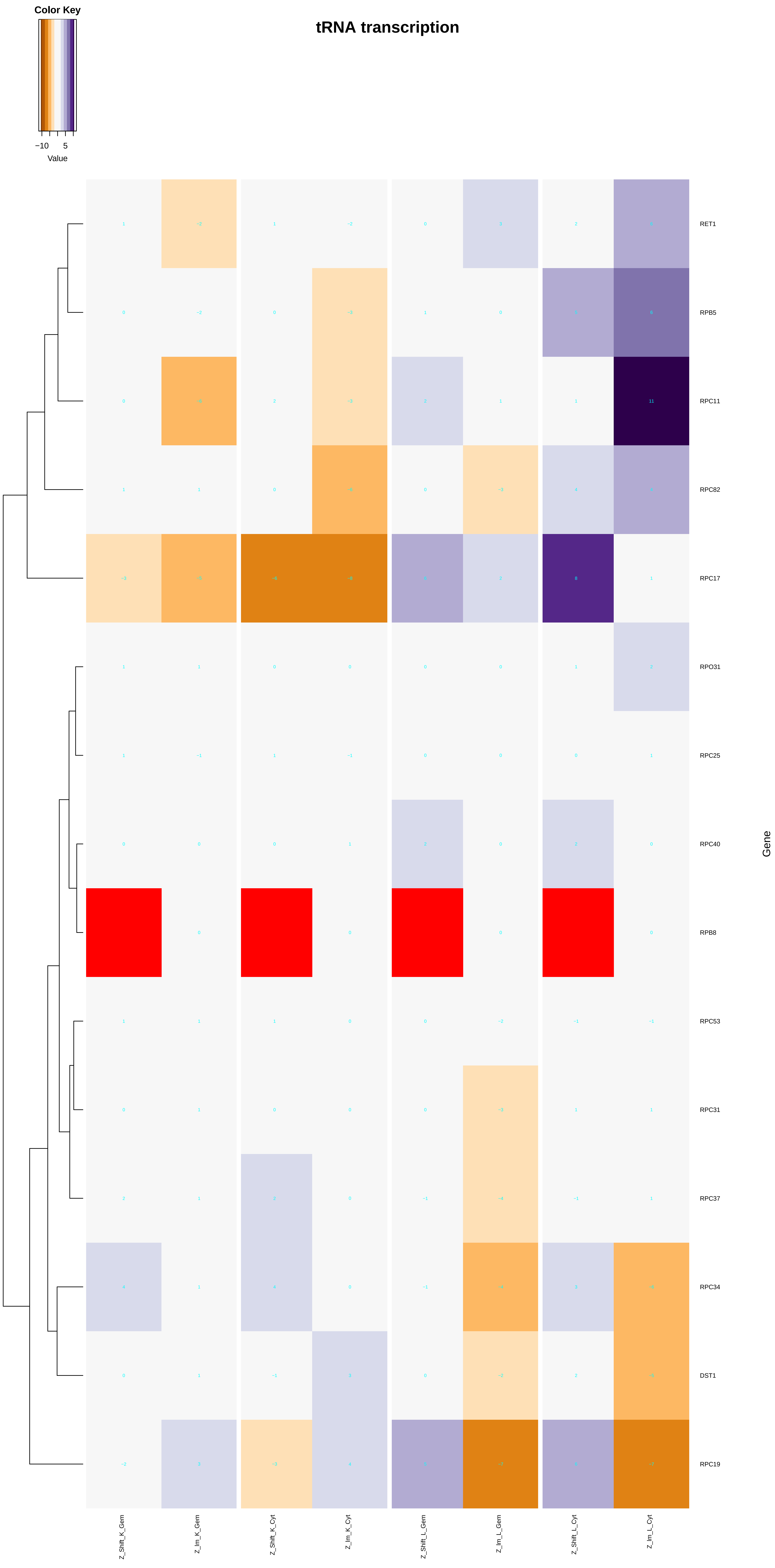
Color Key
−10
5
Value
tRNA transcription
RET1
RPB5
RPC11
RPC82
RPC17
RPO31
RPC25
RPC40
RPB8
RPC53
RPC31
RPC37
RPC34
DST1
RPC19
Gene
Z_Shift_K_Gem
Z_Im_K_Gem
Z_Shift_K_Cyt
Z_Im_K_Cyt
Z_Shift_L_Gem
Z_Im_L_Gem
Z_Shift_L_Cyt
Z_Im_L_Cyt

tRNA catabolic process

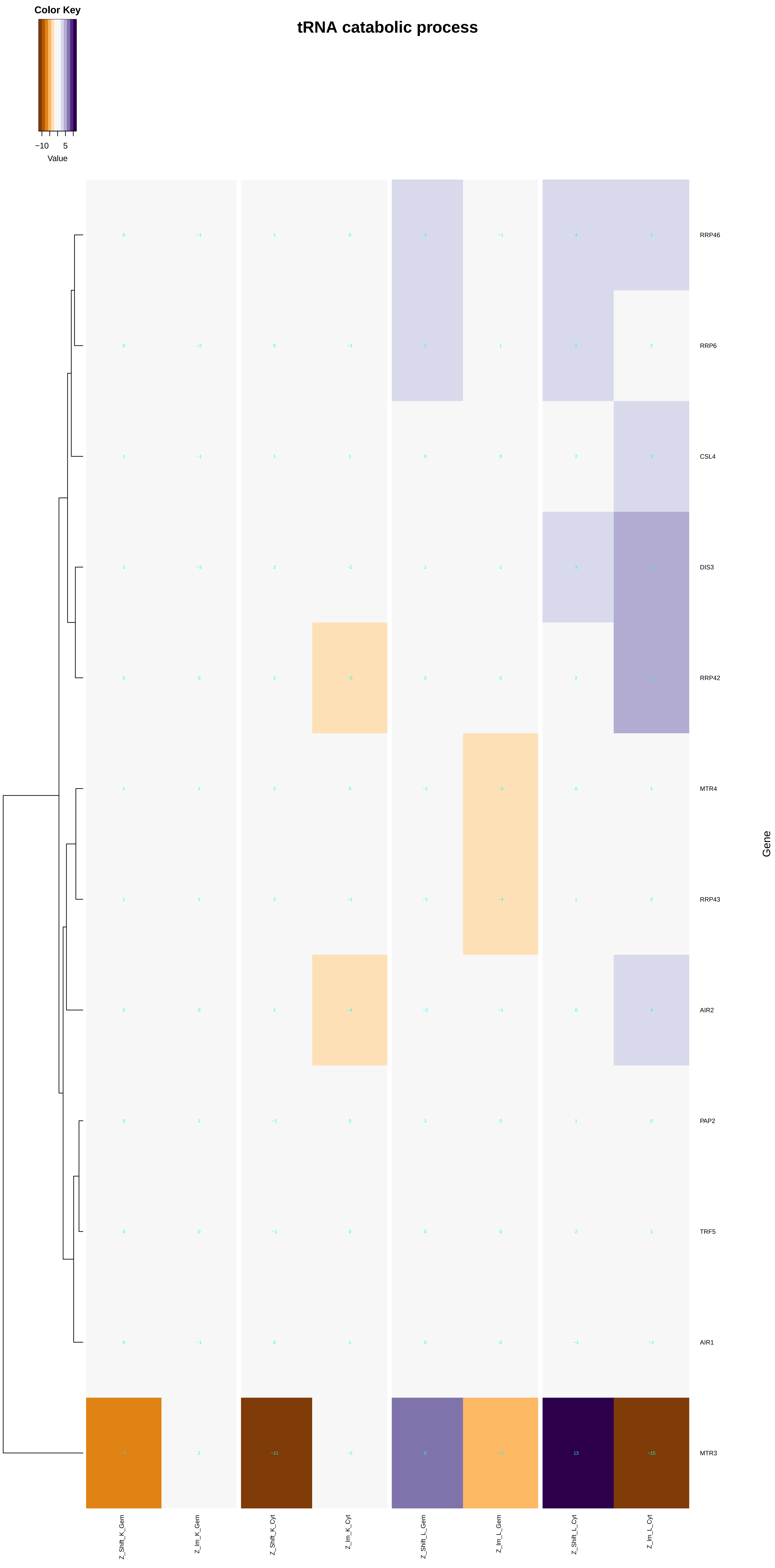

tRNA aminoacylation

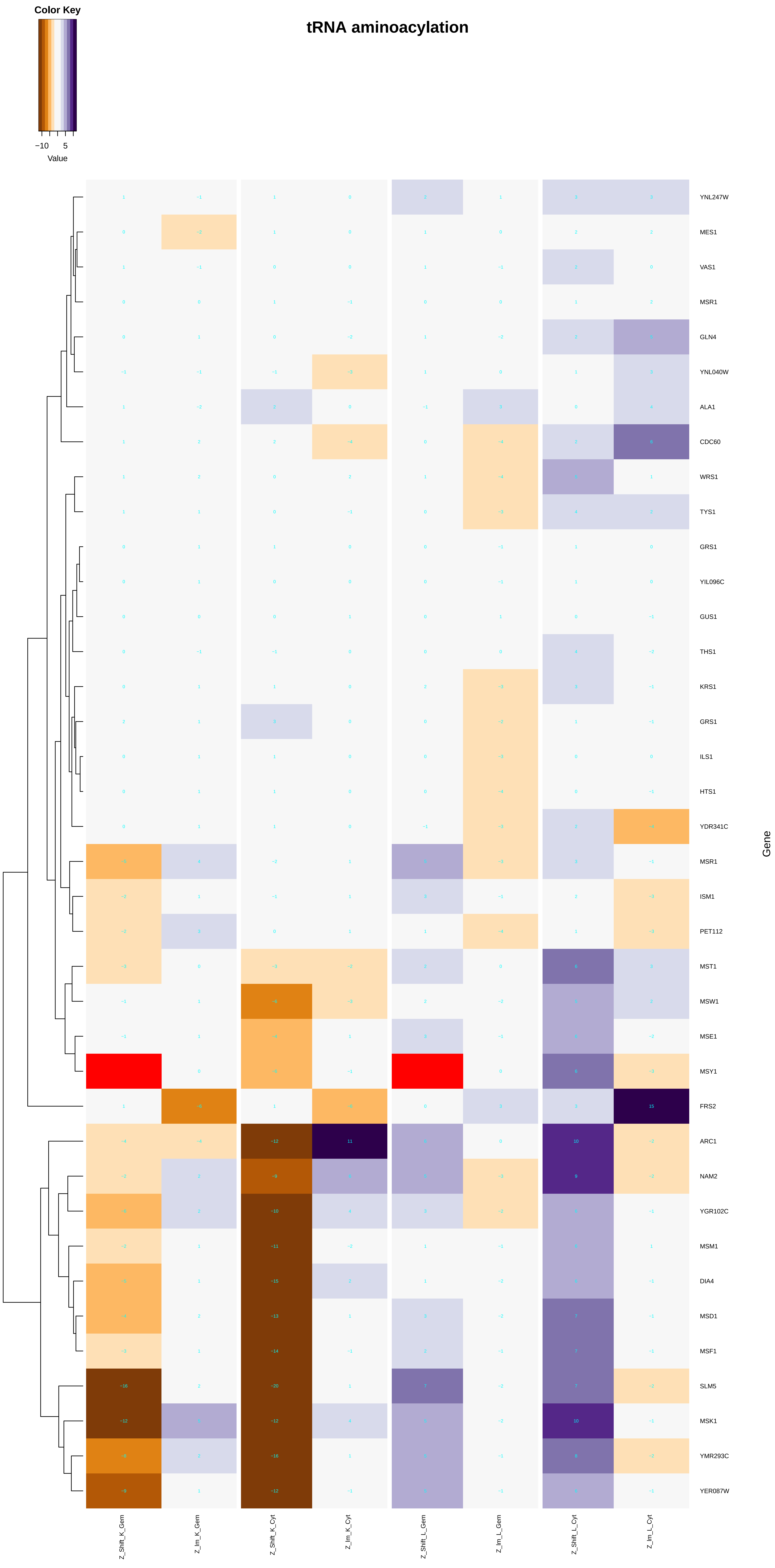

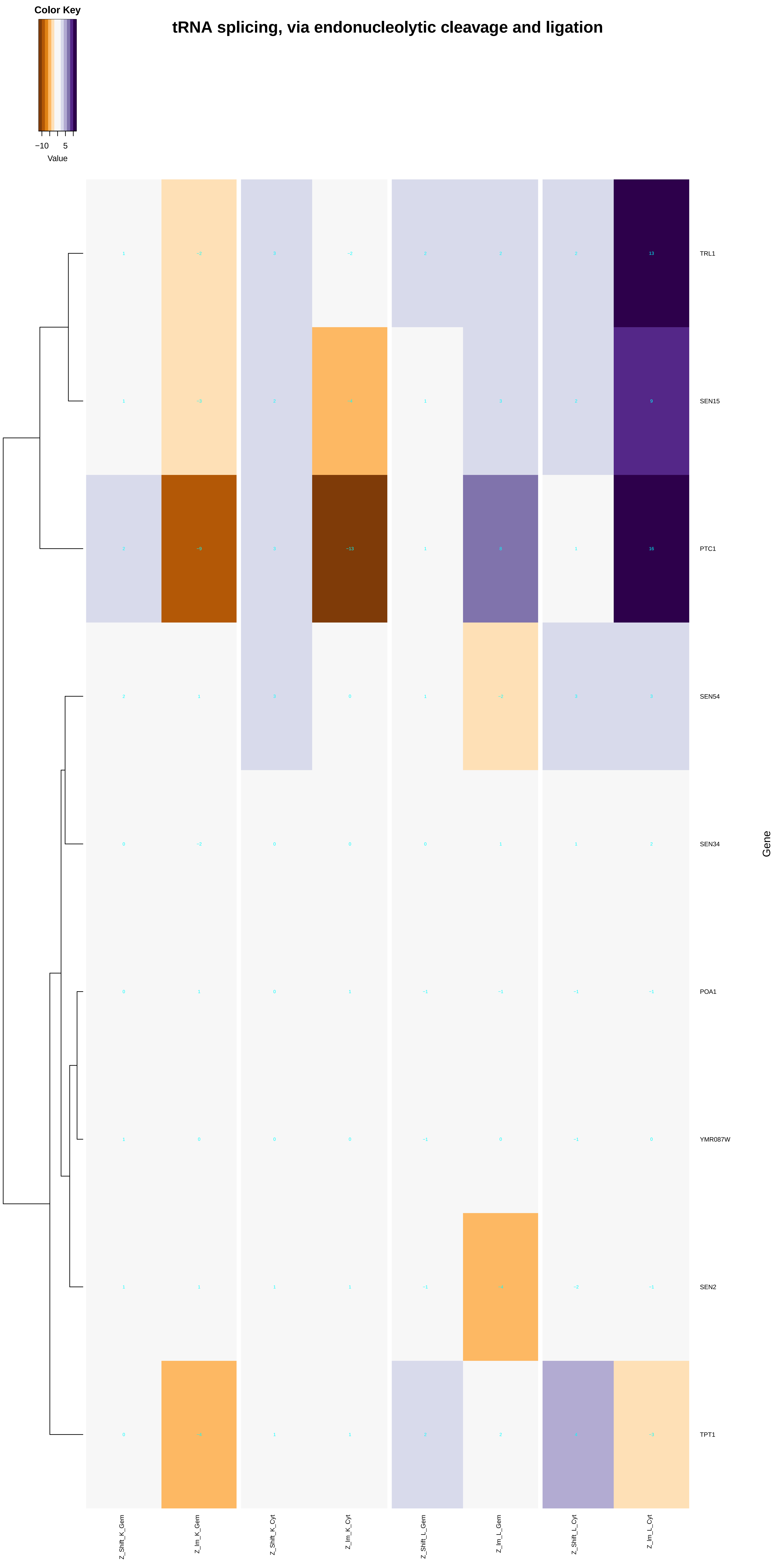

Color Key
−10
5
Value
tRNA splicing, via endonucleolytic cleavage and ligation
TRL1
SEN15
PTC1
SEN54
SEN34
POA1
YMR087W
SEN2
TPT1
Gene
Z_Shift_K_Gem
Z_lm_K_Gem
Z_Shift_K_Cyt
Z_lm_K_Cyt
Z_Shift_L_Gem
Z_lm_L_Gem
Z_Shift_L_Cyt
Z_lm_L_Cyt

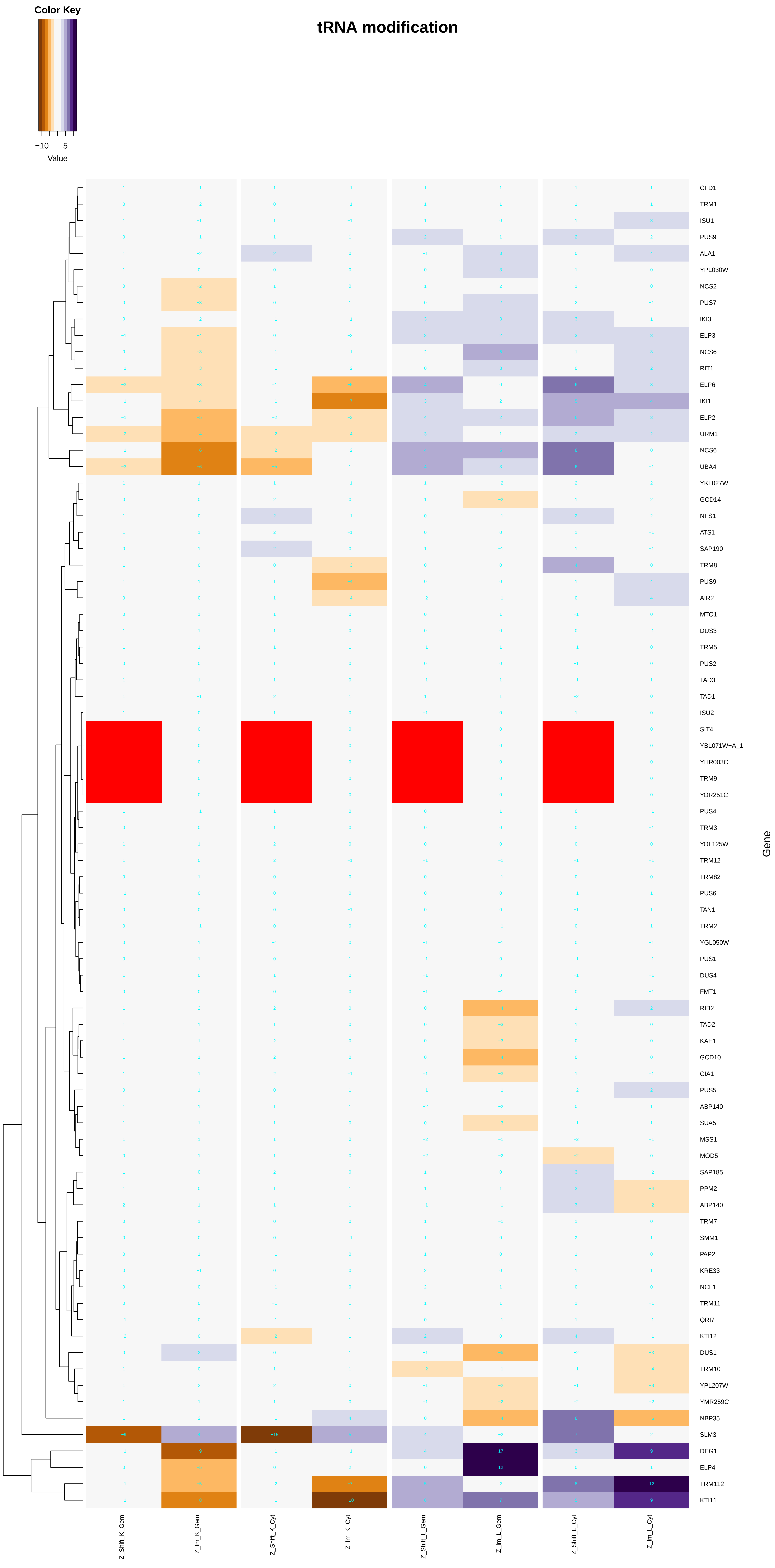
Color Key
−10
5
Value
tRNA modification
CFD1
TRM1
ISU1
PUS9
ALA1
YPL030W
NCS2
PUS7
IKI3
ELP3
NCS6
RIT1
ELP6
IKI1
ELP2
URM1
NCS6
UBA4
YKL027W
GCD14
NFS1
ATS1
SAP190
TRM8
PUS9
AIR2
MTO1
DUS3
TRM5
PUS2
TAD3
TAD1
ISU2
SIT4
YBL071W−A_1
YHR003C
TRM9
YOR251C
PUS4
TRM3
YOL125W
TRM12
TRM82
PUS6
TAN1
TRM2
YGL050W
PUS1
DUS4
FMT1
RIB2
TAD2
KAE1
GCD10
CIA1
PUS5
ABP140
SUA5
MSS1
MOD5
SAP185
PPM2
ABP140
TRM7
SMM1
PAP2
KRE33
NCL1
TRM11
QRI7
KTI12
DUS1
TRM10
YPL207W
YMR259C
NBP35
SLM3
DEG1
ELP4
TRM112
KTI11
Gene
Z_Shift_K_Gem
Z_Im_K_Gem
Z_Shift_K_Cyt
Z_Im_K_Cyt
Z_Shift_L_Gem
Z_Im_L_Gem
Z_Shift_L_Cyt
Z_Im_L_Cyt

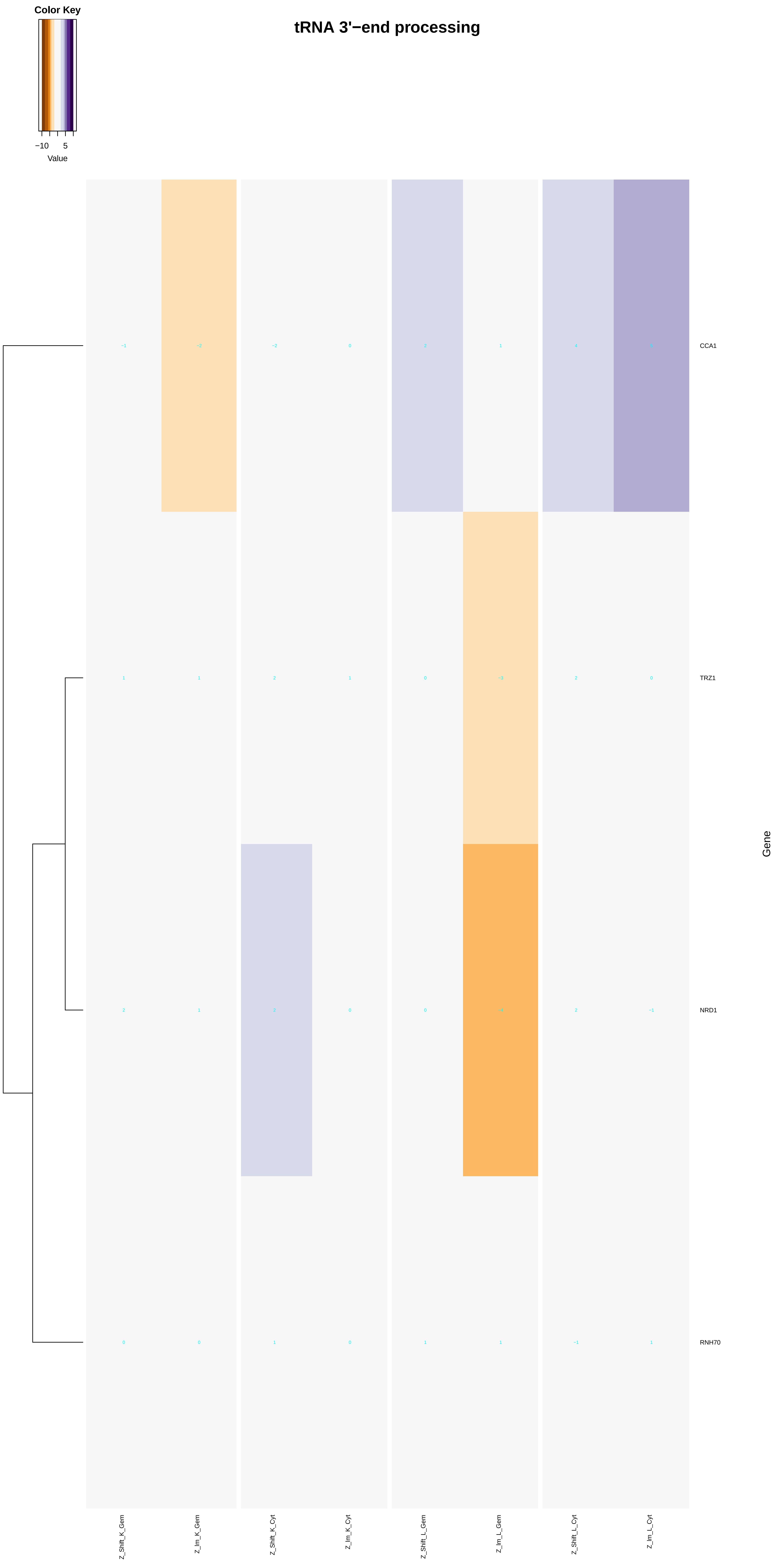
tRNA 3'−end processing
Color Key
−10
5
Value
CCA1
−1
−2
−2
0
2
1
4
5
TRZ1
1
1
2
1
0
−3
2
0
NRD1
2
1
2
0
0
−4
2
−1
RNH70
0
0
1
0
1
1
−1
1
Gene
Z_Shift_K_Gem
Z_lm_K_Gem
Z_Shift_K_Cyt
Z_lm_K_Cyt
Z_Shift_L_Gem
Z_lm_L_Gem
Z_Shift_L_Cyt
Z_lm_L_Cyt

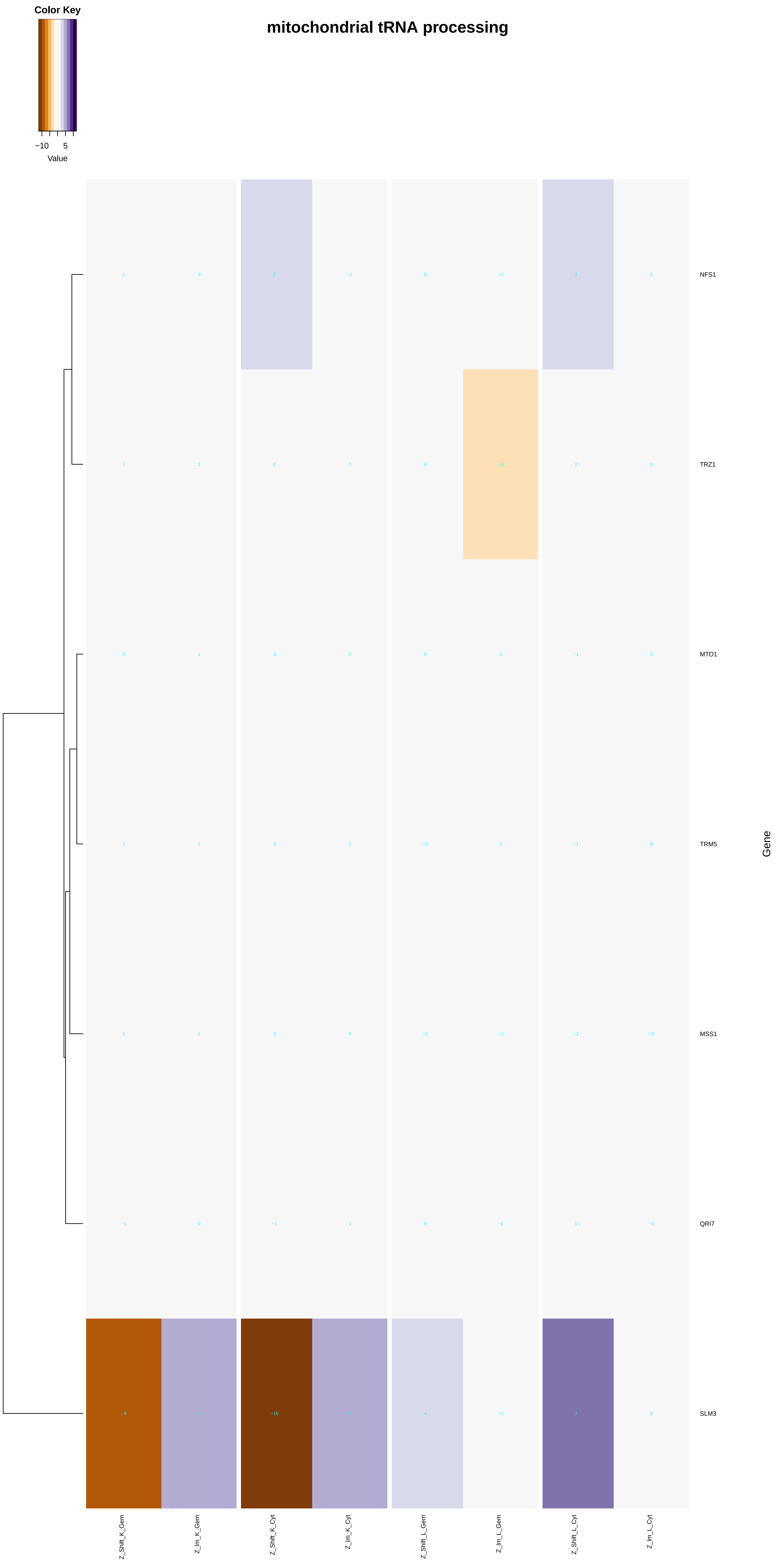

Color Key
−10
5
Value
mitochondrial tRNA processing
NFS1
TRZ1
MTO1
TRM5
MSS1
QRI7
SLM3
Gene
Z_Shift_K_Gem
Z_Im_K_Gem
Z_Shift_K_Cyt
Z_Im_K_Cyt
Z_Shift_L_Gem
Z_Im_L_Gem
Z_Shift_L_Cyt
Z_Im_L_Cyt

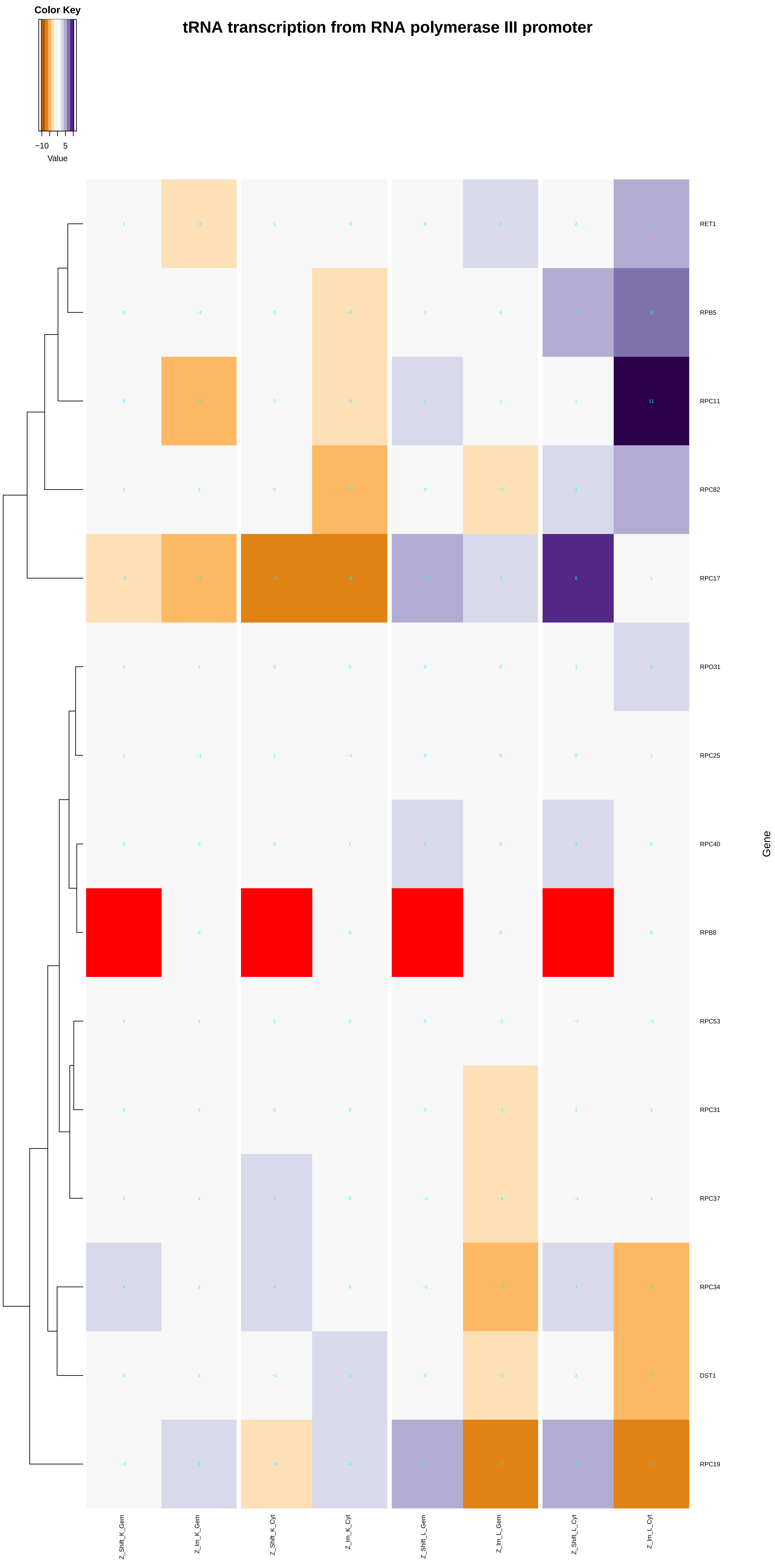

Color Key
−10
5
Value
tRNA transcription from RNA polymerase III promoter
RET1
RPB5
RPC11
RPC82
RPC17
RPO31
RPC25
RPC40
RPB8
RPC53
RPC31
RPC37
RPC34
DST1
RPC19
Gene
Z_Shift_K_Gem
Z_Im_K_Gem
Z_Shift_K_Cyt
Z_Im_K_Cyt
Z_Shift_L_Gem
Z_Im_L_Gem
Z_Shift_L_Cyt
Z_Im_L_Cyt

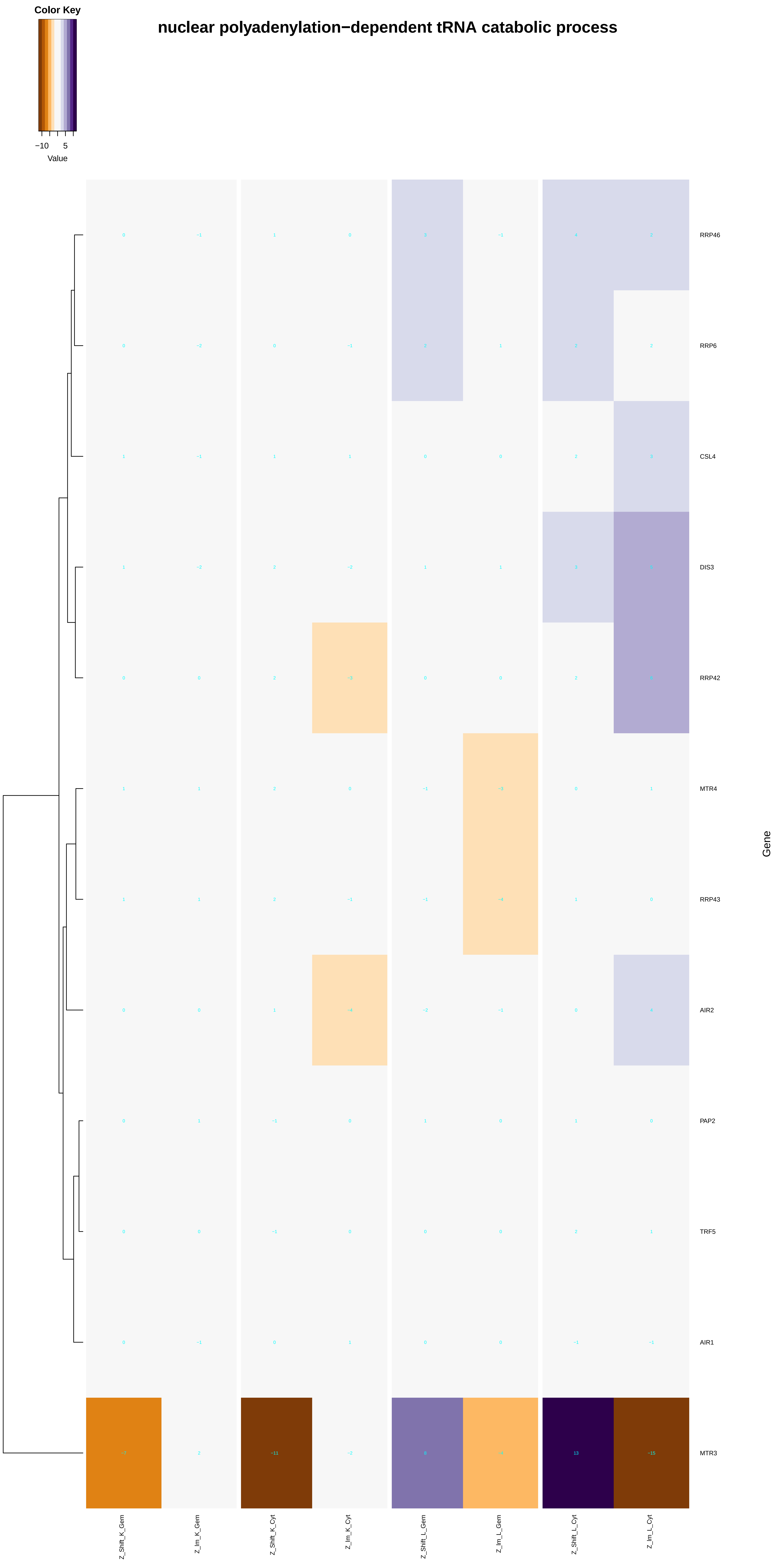

nuclear polyadenylation−dependent tRNA catabolic process
Color Key
−10
5
Value
RRP46
RRP6
CSL4
DIS3
RRP42
MTR4
RRP43
AIR2
PAP2
TRF5
AIR1
MTR3
Gene
Z_Shift_K_Gem
Z_Im_K_Gem
Z_Shift_K_Cyt
Z_Im_K_Cyt
Z_Shift_L_Gem
Z_Im_L_Gem
Z_Shift_L_Cyt
Z_Im_L_Cyt

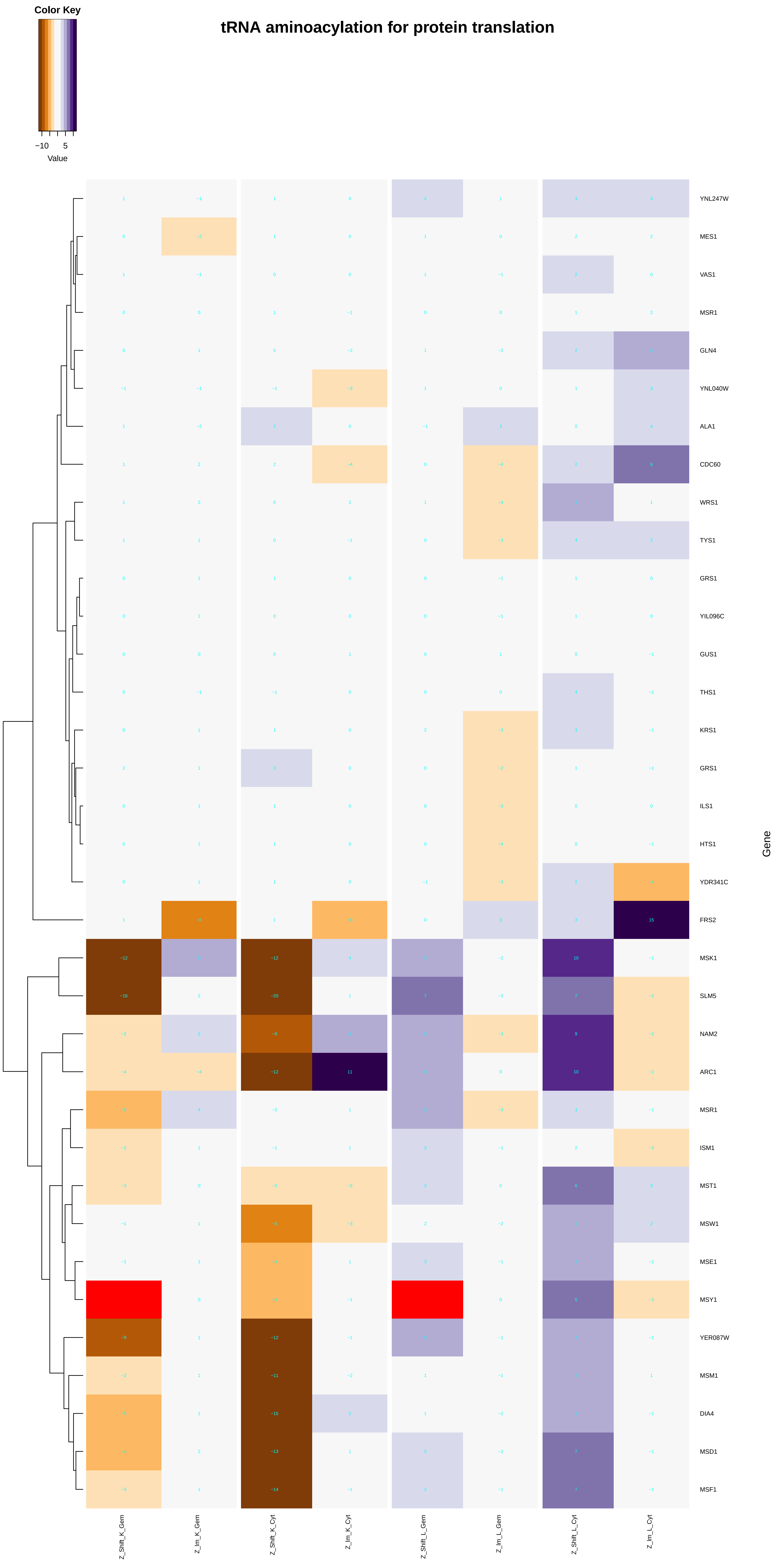
tRNA aminoacylation for protein translation
Color Key
−10
5
Value
YNL247W
MES1
VAS1
MSR1
GLN4
YNL040W
ALA1
CDC60
WRS1
TYS1
GRS1
YIL096C
GUS1
THS1
KRS1
GRS1
ILS1
HTS1
YDR341C
FRS2
MSK1
SLM5
NAM2
ARC1
MSR1
ISM1
MST1
MSW1
MSE1
MSY1
YER087W
MSM1
DIA4
MSD1
MSF1
Gene
Z_Shift_K_Gem
Z_Im_K_Gem
Z_Shift_K_Cyt
Z_Im_K_Cyt
Z_Shift_L_Gem
Z_Im_L_Gem
Z_Shift_L_Cyt
Z_Im_L_Cyt

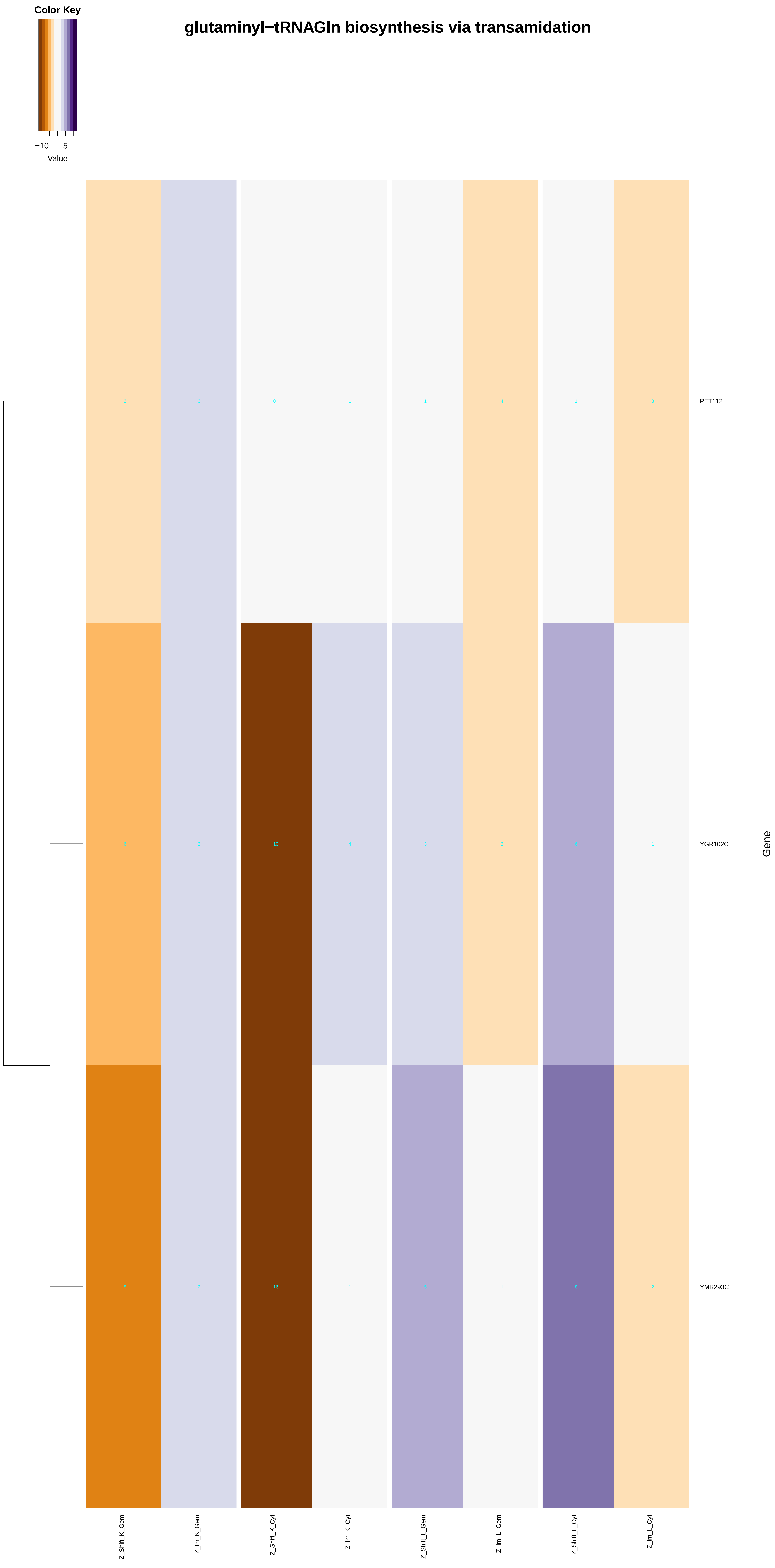
Color Key
−10
5
Value
glutaminyl−tRNAGln biosynthesis via transamidation
PET112
YGR102C
YMR293C
Gene
Z_Shift_K_Gem
Z_Im_K_Gem
Z_Shift_K_Cyt
Z_Im_K_Cyt
Z_Shift_L_Gem
Z_Im_L_Gem
Z_Shift_L_Cyt
Z_Im_L_Cyt

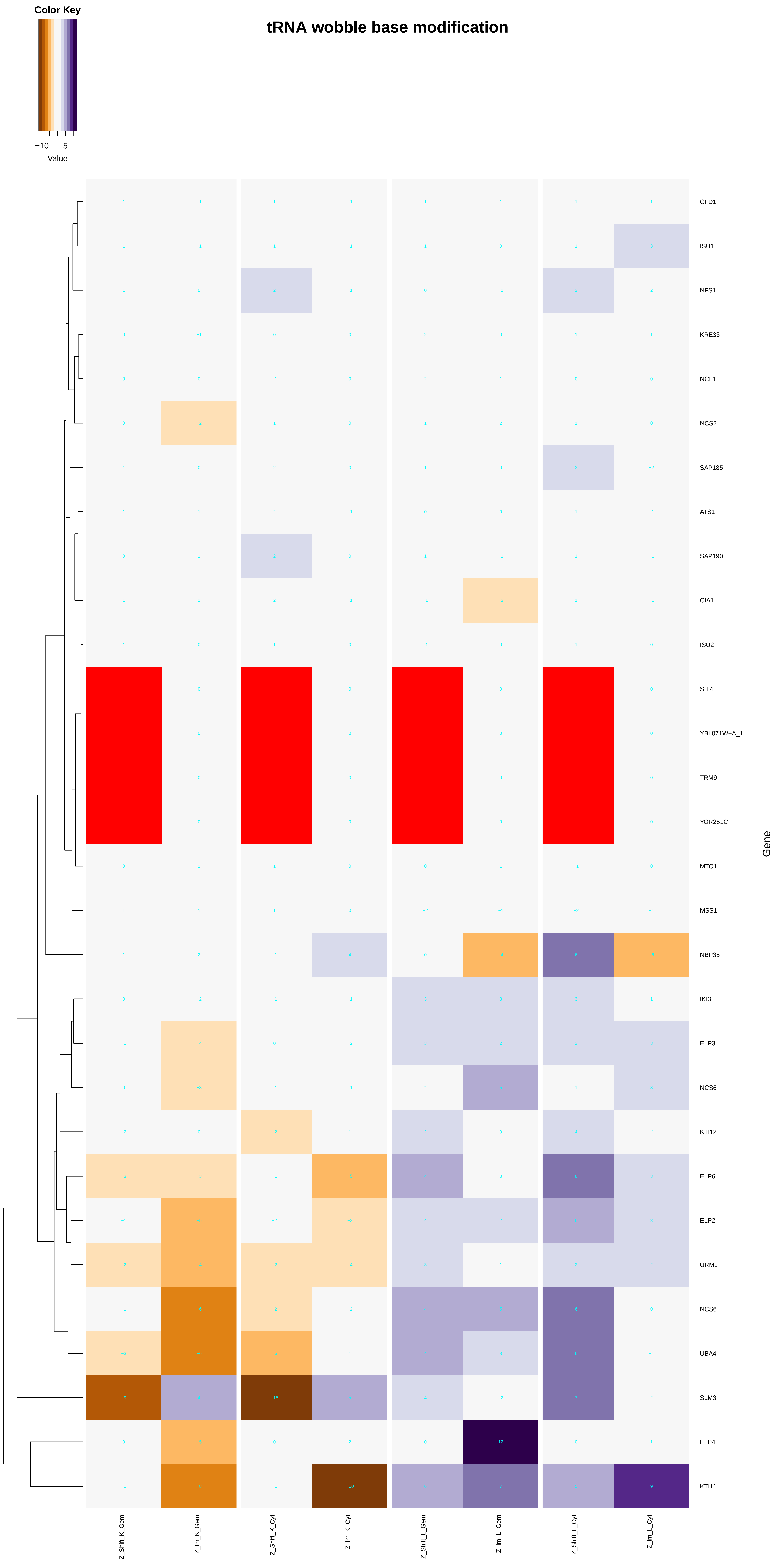

Color Key
−10
5
Value
tRNA wobble base modification
CFD1
ISU1
NFS1
KRE33
NCL1
NCS2
SAP185
ATS1
SAP190
CIA1
ISU2
SIT4
YBL071W−A_1
TRM9
YOR251C
MTO1
MSS1
NBP35
IKI3
ELP3
NCS6
KTI12
ELP6
ELP2
URM1
NCS6
UBA4
SLM3
ELP4
KTI11
Gene
Z_Shift_K_Gem
Z_Im_K_Gem
Z_Shift_K_Cyt
Z_Im_K_Cyt
Z_Shift_L_Gem
Z_Im_L_Gem
Z_Shift_L_Cyt
Z_Im_L_Cyt

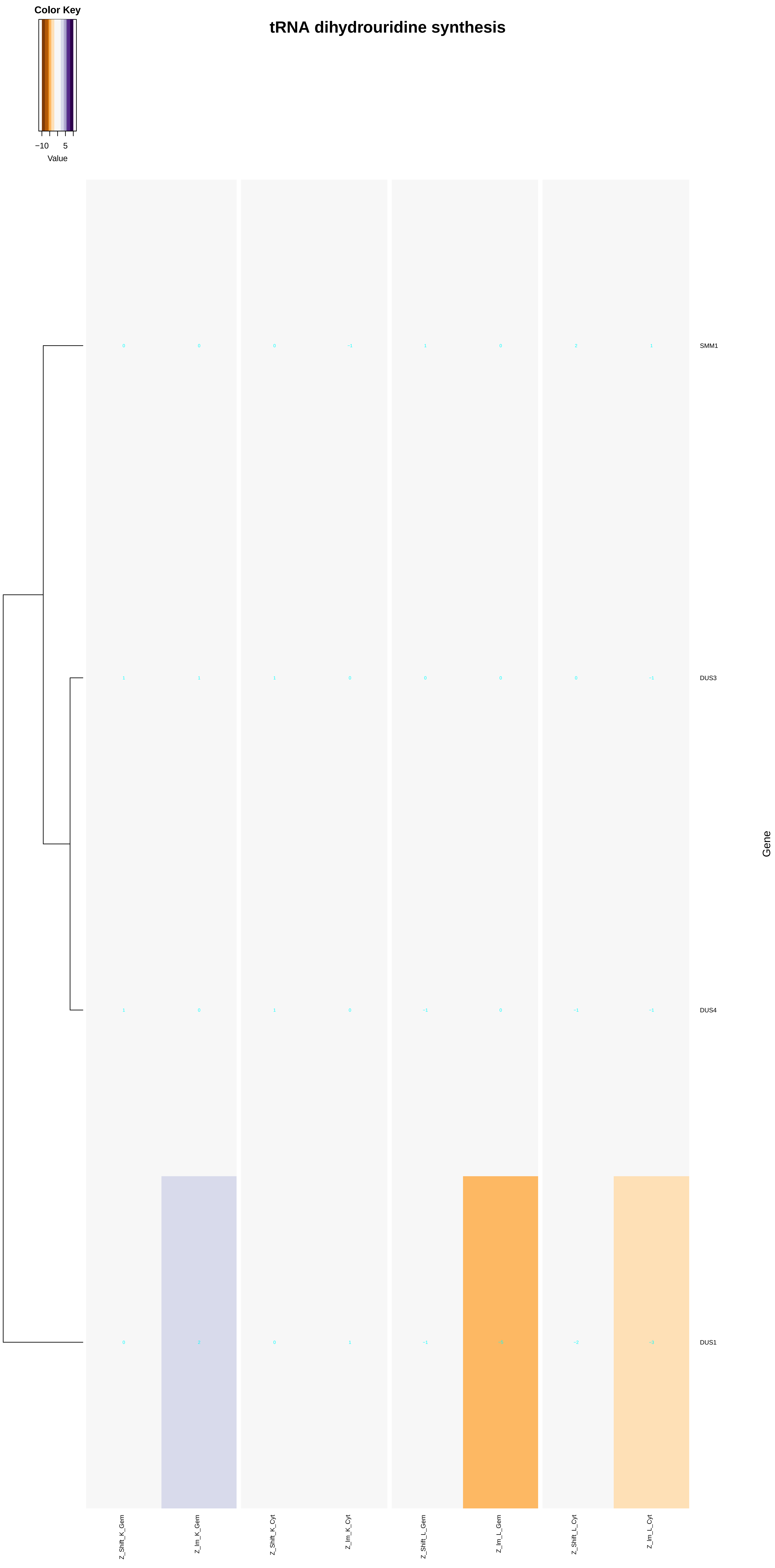

tRNA dihydrouridine synthesis
Color Key
−10
5
Value
SMM1
DUS3
DUS4
DUS1
Gene
Z_Shift_K_Gem
Z_Im_K_Gem
Z_Shift_K_Cyt
Z_Im_K_Cyt
Z_Shift_L_Gem
Z_Im_L_Gem
Z_Shift_L_Cyt
Z_Im_L_Cyt

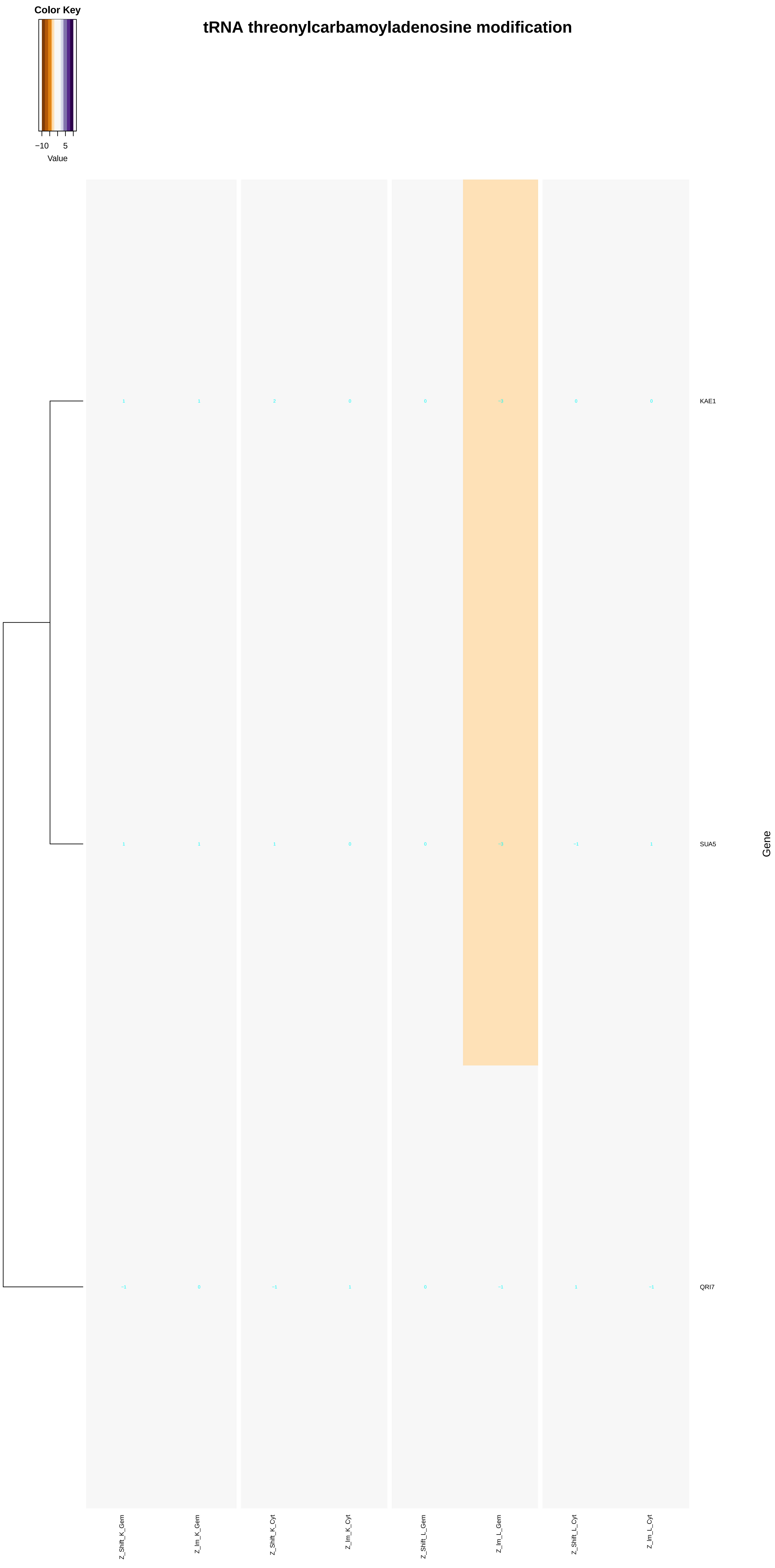
tRNA threonylcarbamoyladenosine modification
Color Key
−10
5
Value
KAE1
SUA5
QRI7
Gene
Z_Shift_K_Gem
Z_Im_K_Gem
Z_Shift_K_Cyt
Z_Im_K_Cyt
Z_Shift_L_Gem
Z_Im_L_Gem
Z_Shift_L_Cyt
Z_Im_L_Cyt
1 1 2 0 0 −3 0 0
1 1 1 0 0 −3 −1 1
−1 0 −1 1 0 −1 1 −1

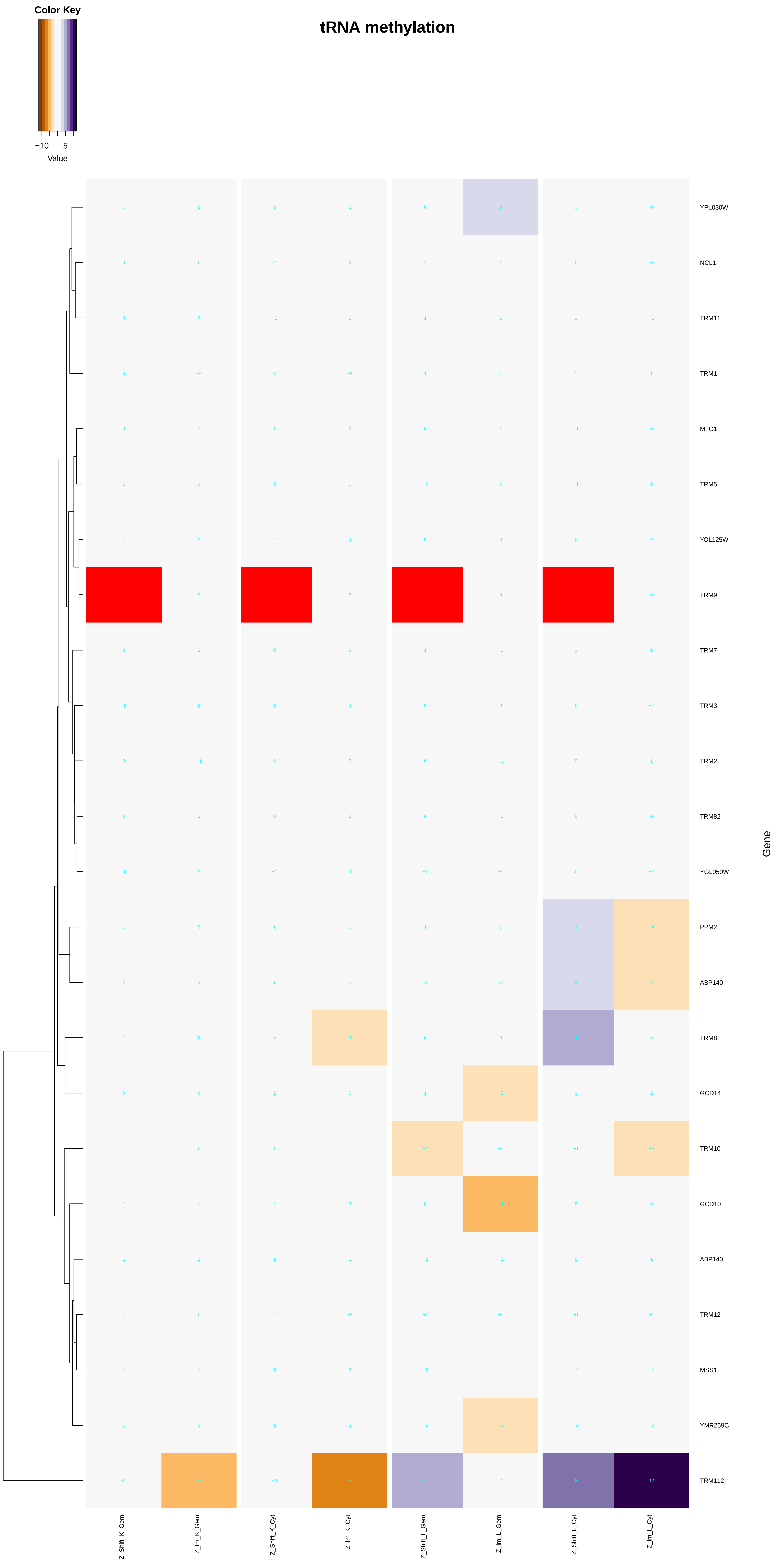

Color Key
−10
5
Value
tRNA methylation
YPL030W
NCL1
TRM11
TRM1
MTO1
TRM5
YOL125W
TRM9
TRM7
TRM3
TRM2
TRM82
YGL050W
PPM2
ABP140
TRM8
GCD14
TRM10
GCD10
ABP140
TRM12
MSS1
YMR259C
TRM112
Gene
Z_Shift_K_Gem
Z_lm_K_Gem
Z_Shift_K_Cyt
Z_lm_K_Cyt
Z_Shift_L_Gem
Z_lm_L_Gem
Z_Shift_L_Cyt
Z_lm_L_Cyt

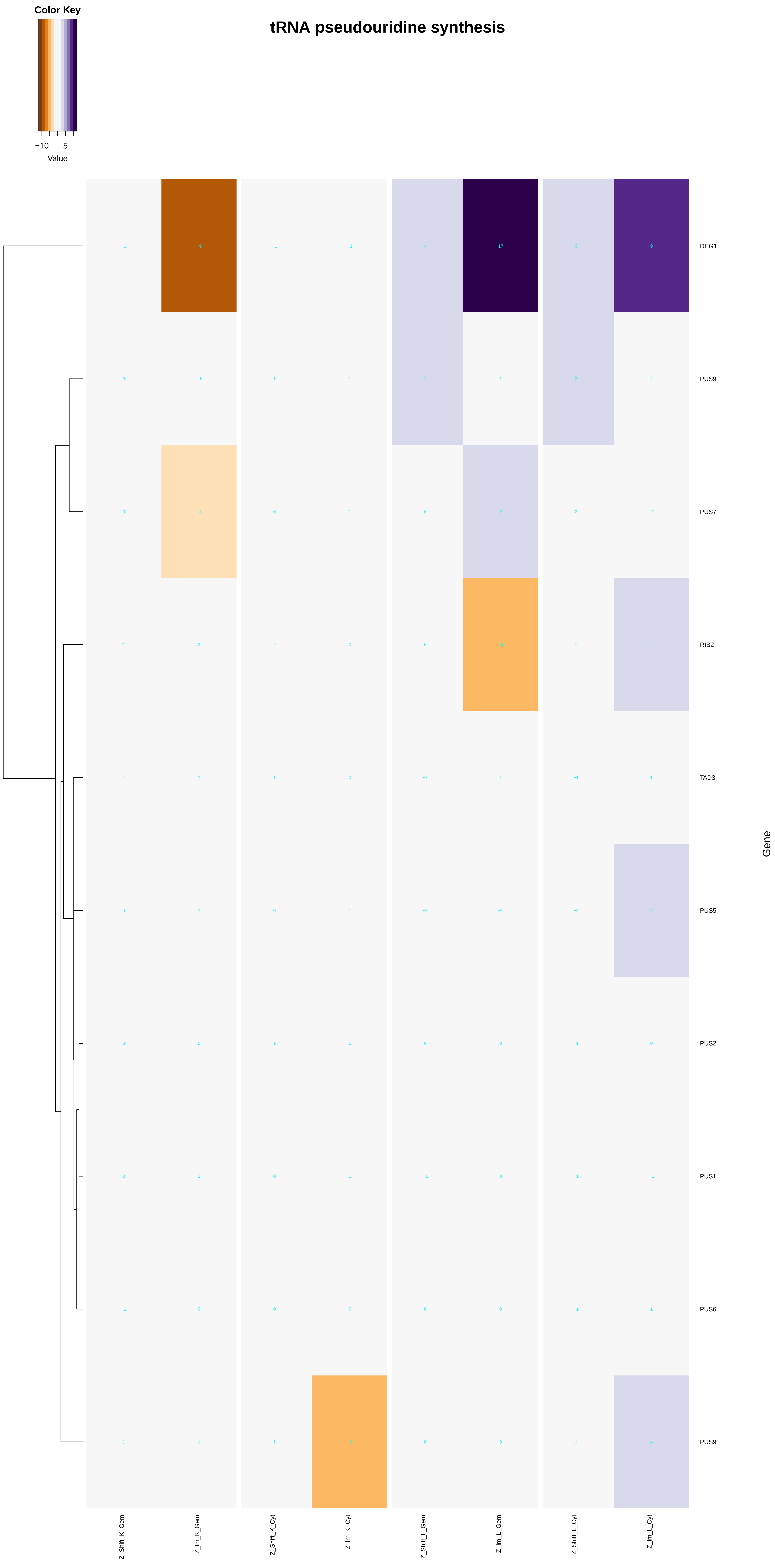
Color Key
−10
5
Value
tRNA pseudouridine synthesis
DEG1
PUS9
PUS7
RIB2
TAD3
PUS5
PUS2
PUS1
PUS6
PUS9
Gene
Z_Shift_K_Gem
Z_Im_K_Gem
Z_Shift_K_Cyt
Z_Im_K_Cyt
Z_Shift_L_Gem
Z_Im_L_Gem
Z_Shift_L_Cyt
Z_Im_L_Cyt

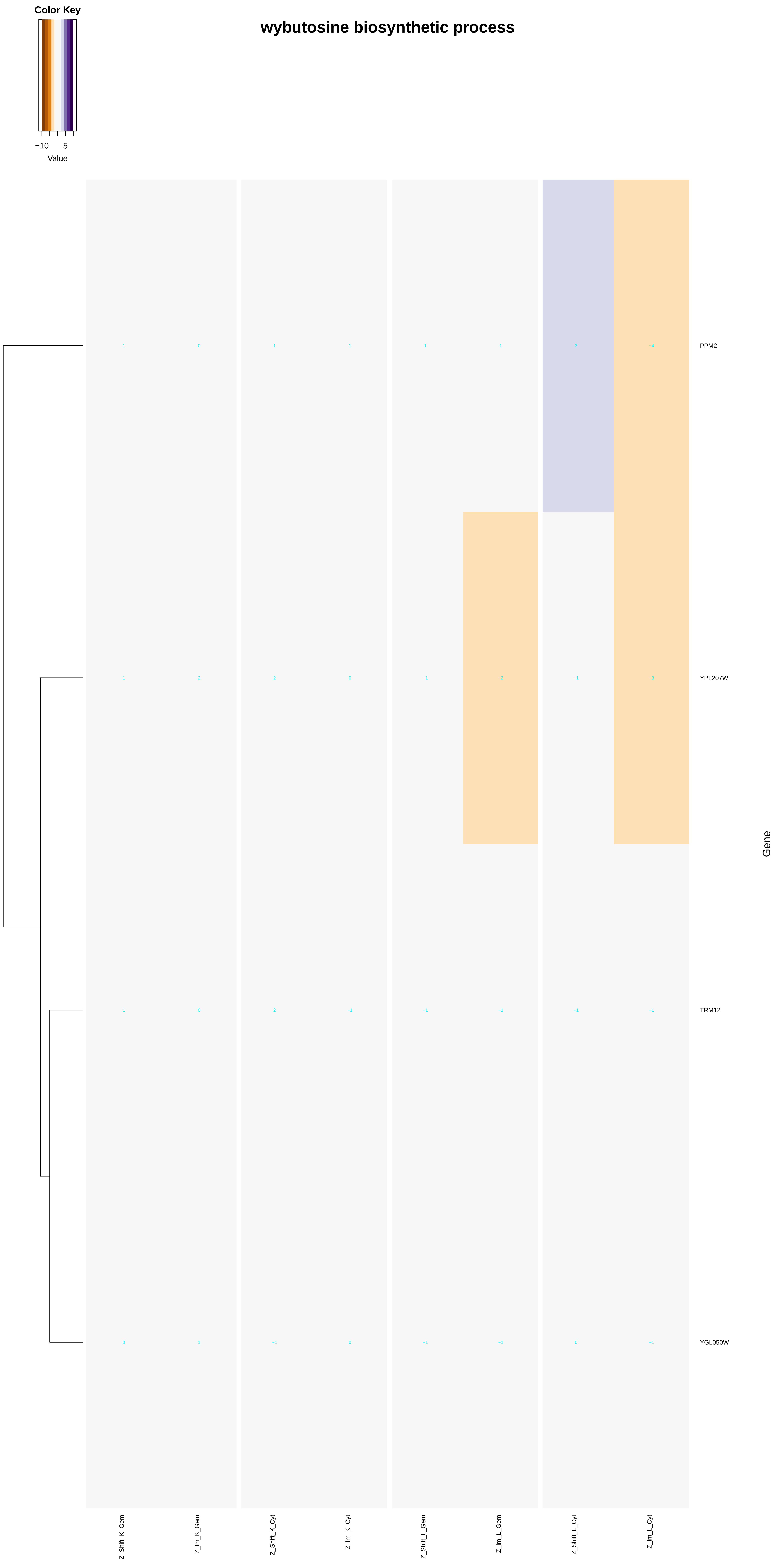

Color Key
−10
5
Value
wybutosine biosynthetic process
PPM2
YPL207W
TRM12
YGL050W
Gene
Z_Shift_K_Gem
Z_Im_K_Gem
Z_Shift_K_Cyt
Z_Im_K_Cyt
Z_Shift_L_Gem
Z_Im_L_Gem
Z_Shift_L_Cyt
Z_Im_L_Cyt

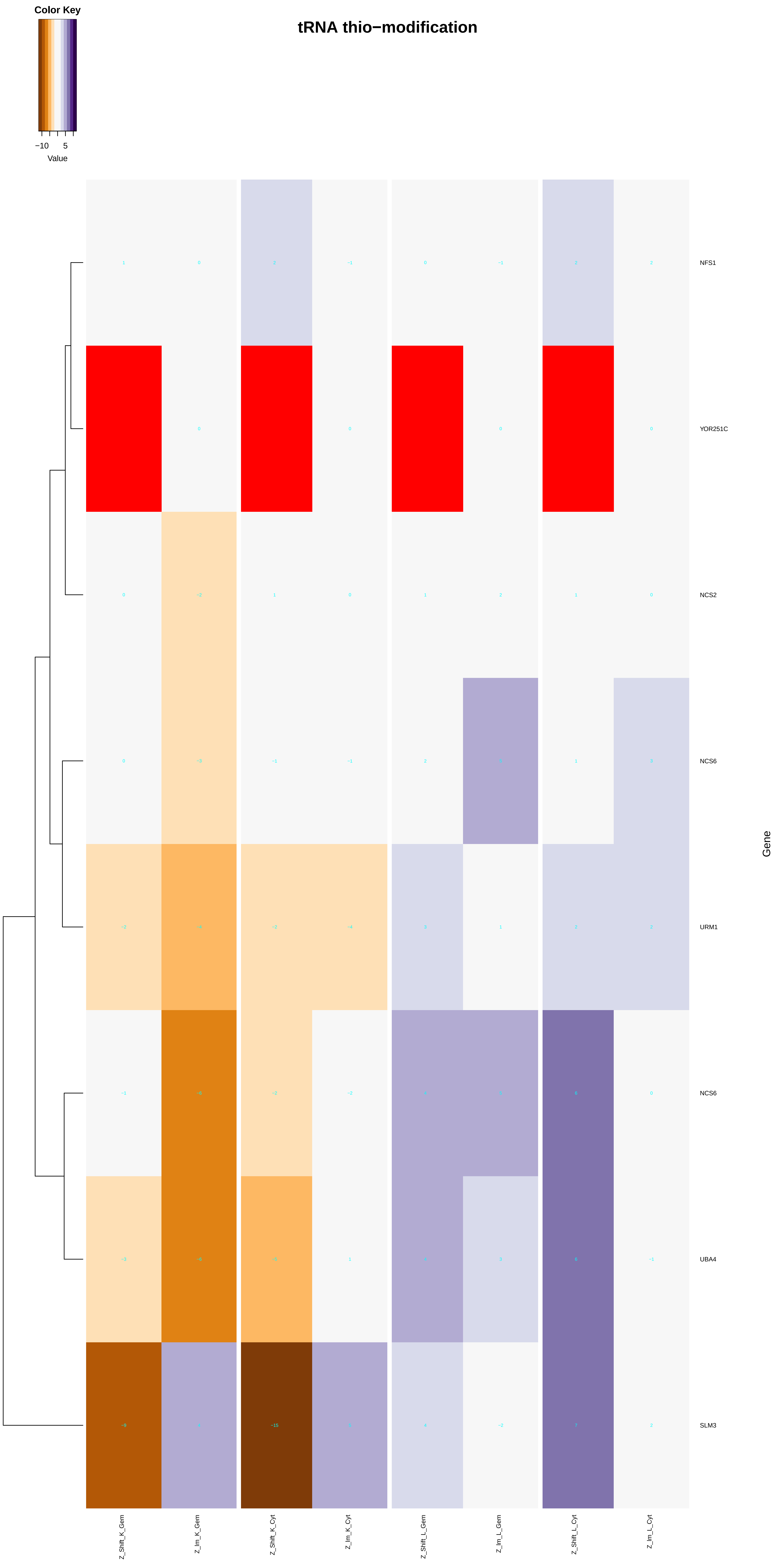

tRNA thio−modification
Color Key
−10
5
Value
NFS1
YOR251C
NCS2
NCS6
URM1
NCS6
UBA4
SLM3
Gene
Z_Shift_K_Gem
Z_Im_K_Gem
Z_Shift_K_Cyt
Z_Im_K_Cyt
Z_Shift_L_Gem
Z_Im_L_Gem
Z_Shift_L_Cyt
Z_Im_L_Cyt

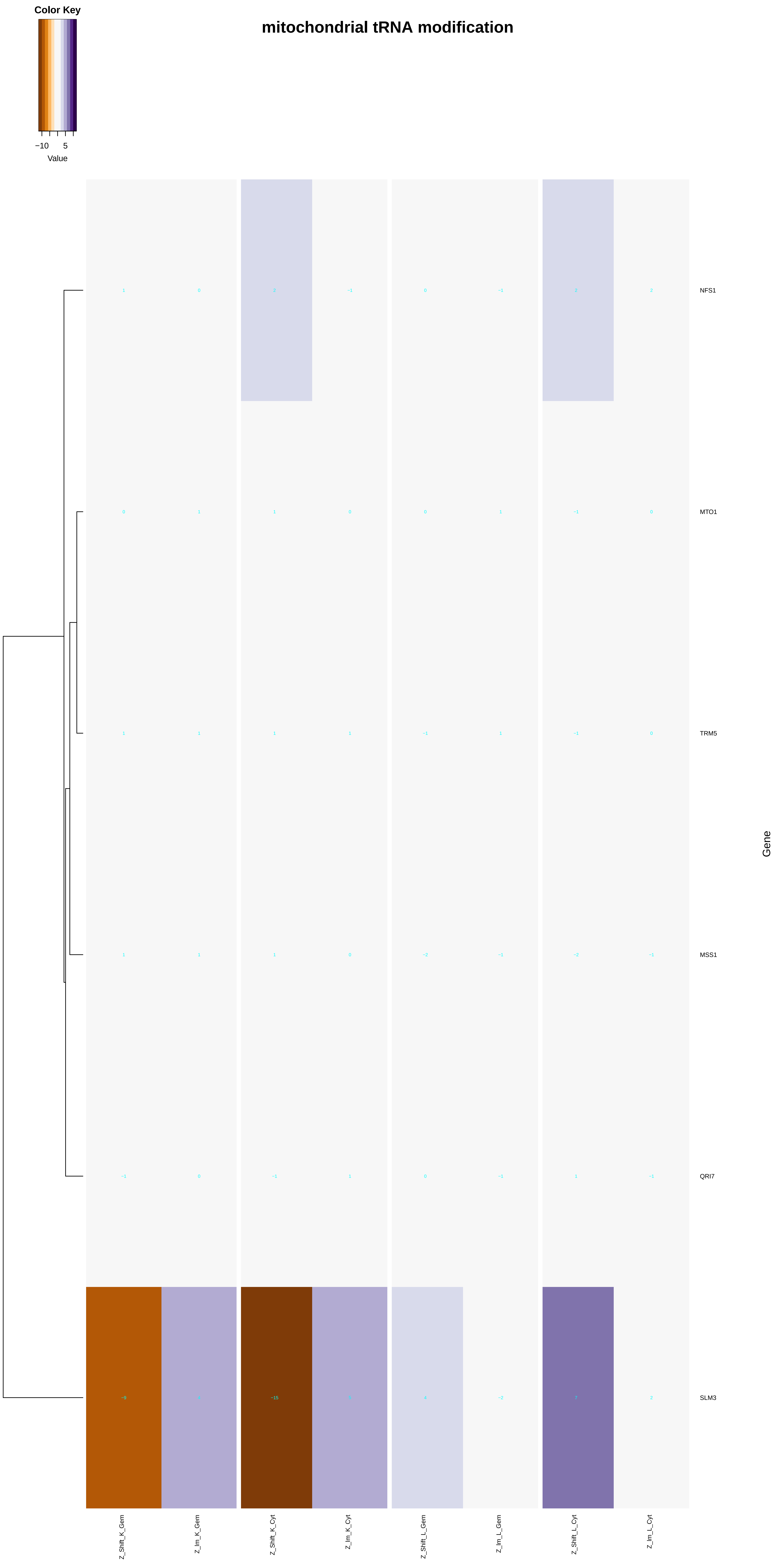
Color Key
−10
5
Value
mitochondrial tRNA modification
NFS1
MTO1
TRM5
MSS1
QRI7
SLM3
Gene
Z_Shift_K_Gem
Z_Im_K_Gem
Z_Shift_K_Cyt
Z_Im_K_Cyt
Z_Shift_L_Gem
Z_Im_L_Gem
Z_Shift_L_Cyt
Z_Im_L_Cyt

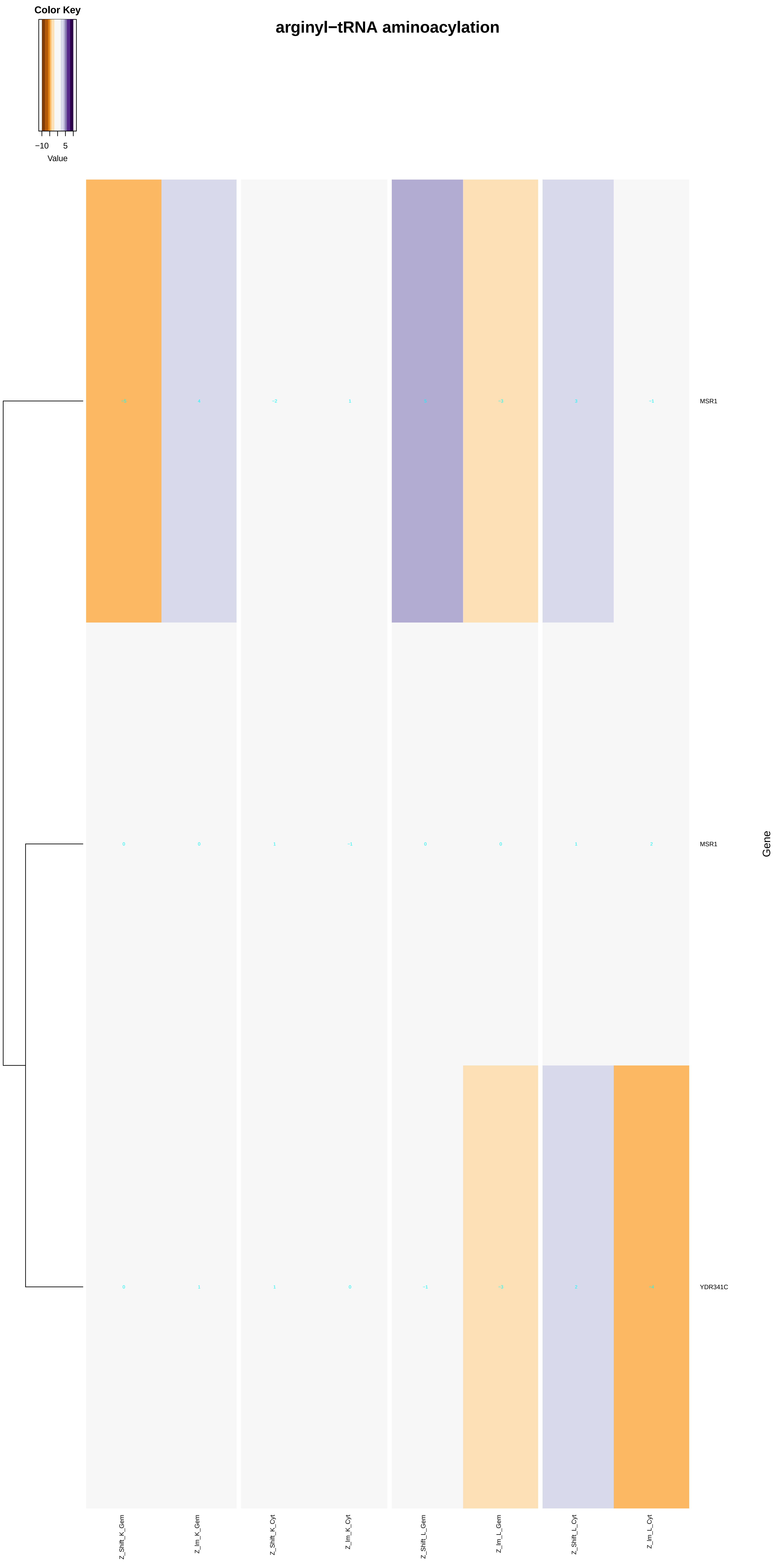

arginyl−tRNA aminoacylation
Color Key
−10
5
Value
MSR1
MSR1
YDR341C
Gene
Z_Shift_K_Gem
Z_Im_K_Gem
Z_Shift_K_Cyt
Z_Im_K_Cyt
Z_Shift_L_Gem
Z_Im_L_Gem
Z_Shift_L_Cyt
Z_Im_L_Cyt

phenylalanyl−tRNA aminoacylation

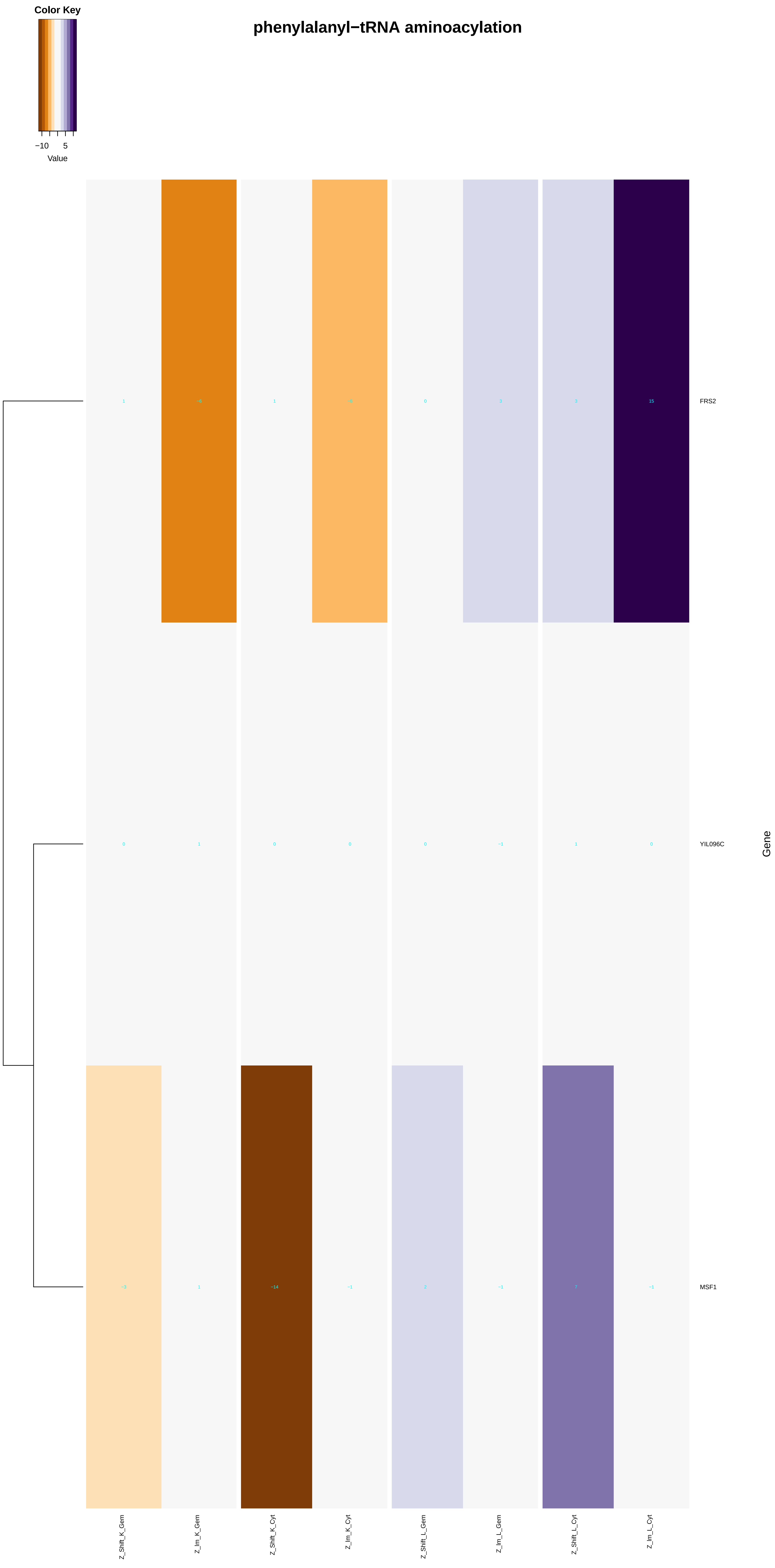

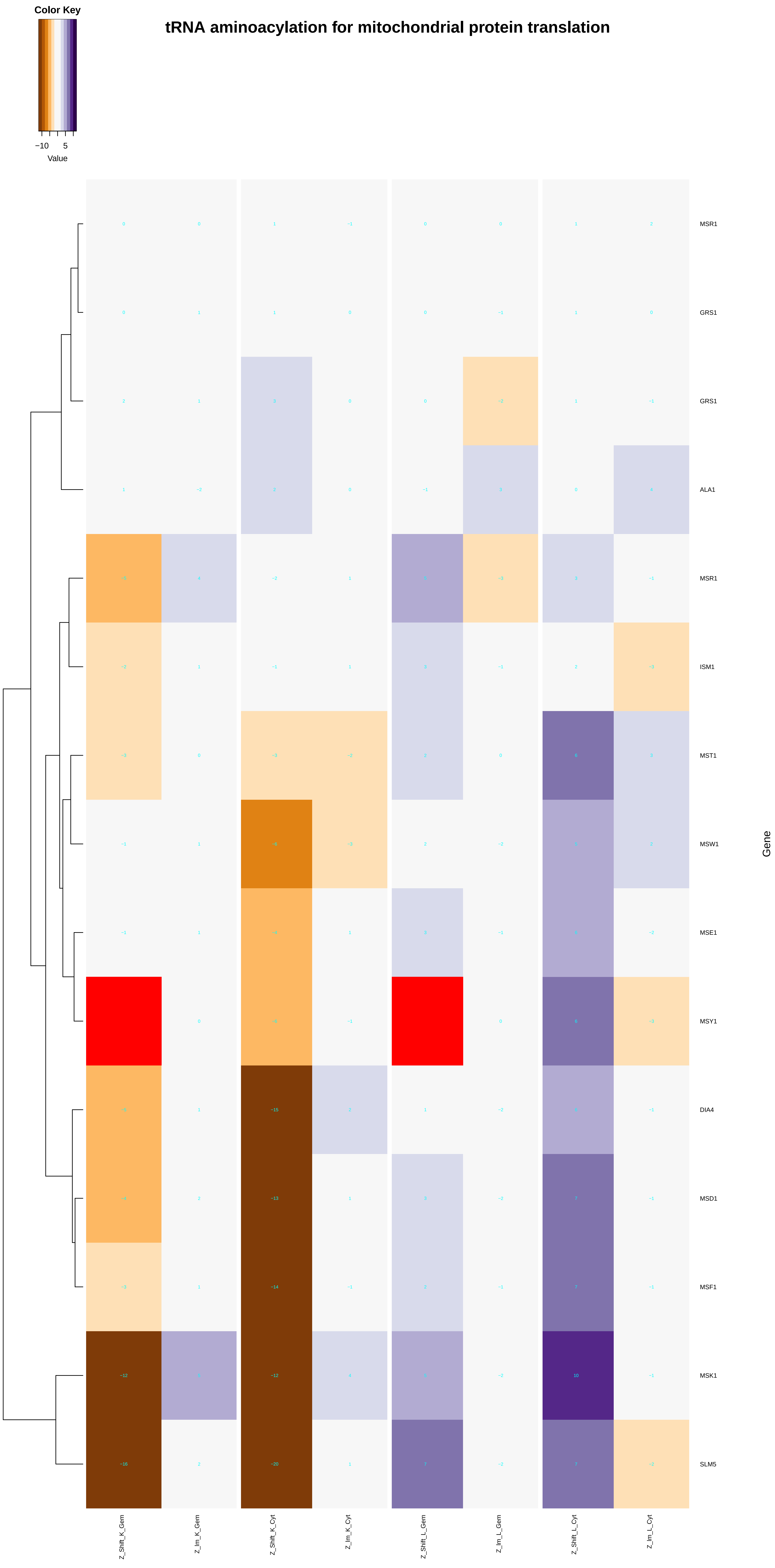
Color Key
−10
5
Value
tRNA aminoacylation for mitochondrial protein translation
MSR1
GRS1
GRS1
ALA1
MSR1
ISM1
MST1
MSW1
MSE1
MSY1
DIA4
MSD1
MSF1
MSK1
SLM5
Gene
Z_Shift_K_Gem
Z_Im_K_Gem
Z_Shift_K_Cyt
Z_Im_K_Cyt
Z_Shift_L_Gem
Z_Im_L_Gem
Z_Shift_L_Cyt
Z_Im_L_Cyt

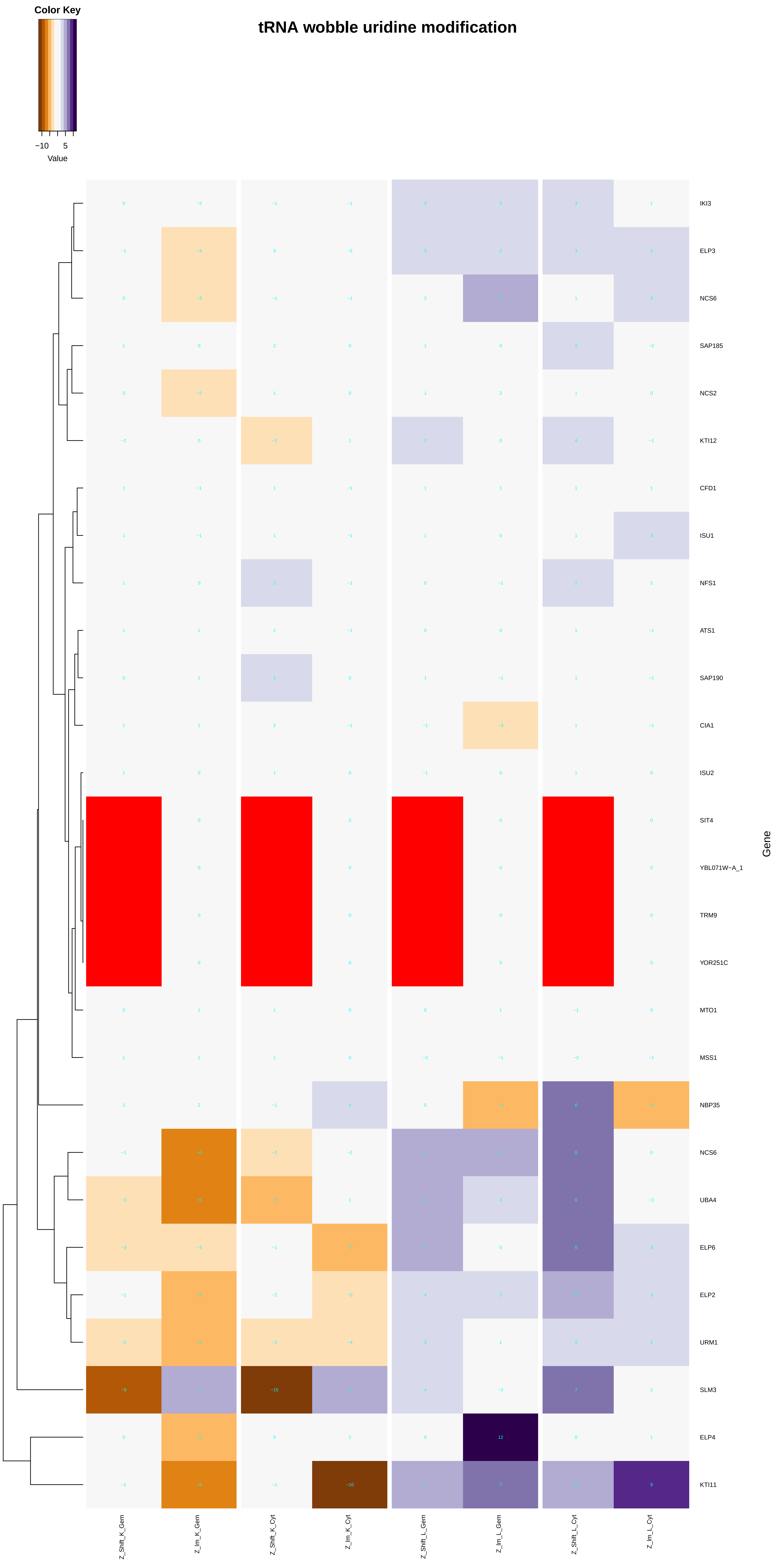
Color Key
−10 5
Value
tRNA wobble uridine modification
IKI3
ELP3
NCS6
SAP185
NCS2
KTI12
CFD1
ISU1
NFS1
ATS1
SAP190
CIA1
ISU2
SIT4
YBL071W−A_1
TRM9
YOR251C
MTO1
MSS1
NBP35
NCS6
UBA4
ELP6
ELP2
URM1
SLM3
ELP4
KTI11
Gene
Z_Shift_K_Gem
Z_Im_K_Gem
Z_Shift_K_Cyt
Z_Im_K_Cyt
Z_Shift_L_Gem
Z_Im_L_Gem
Z_Shift_L_Cyt
Z_Im_L_Cyt

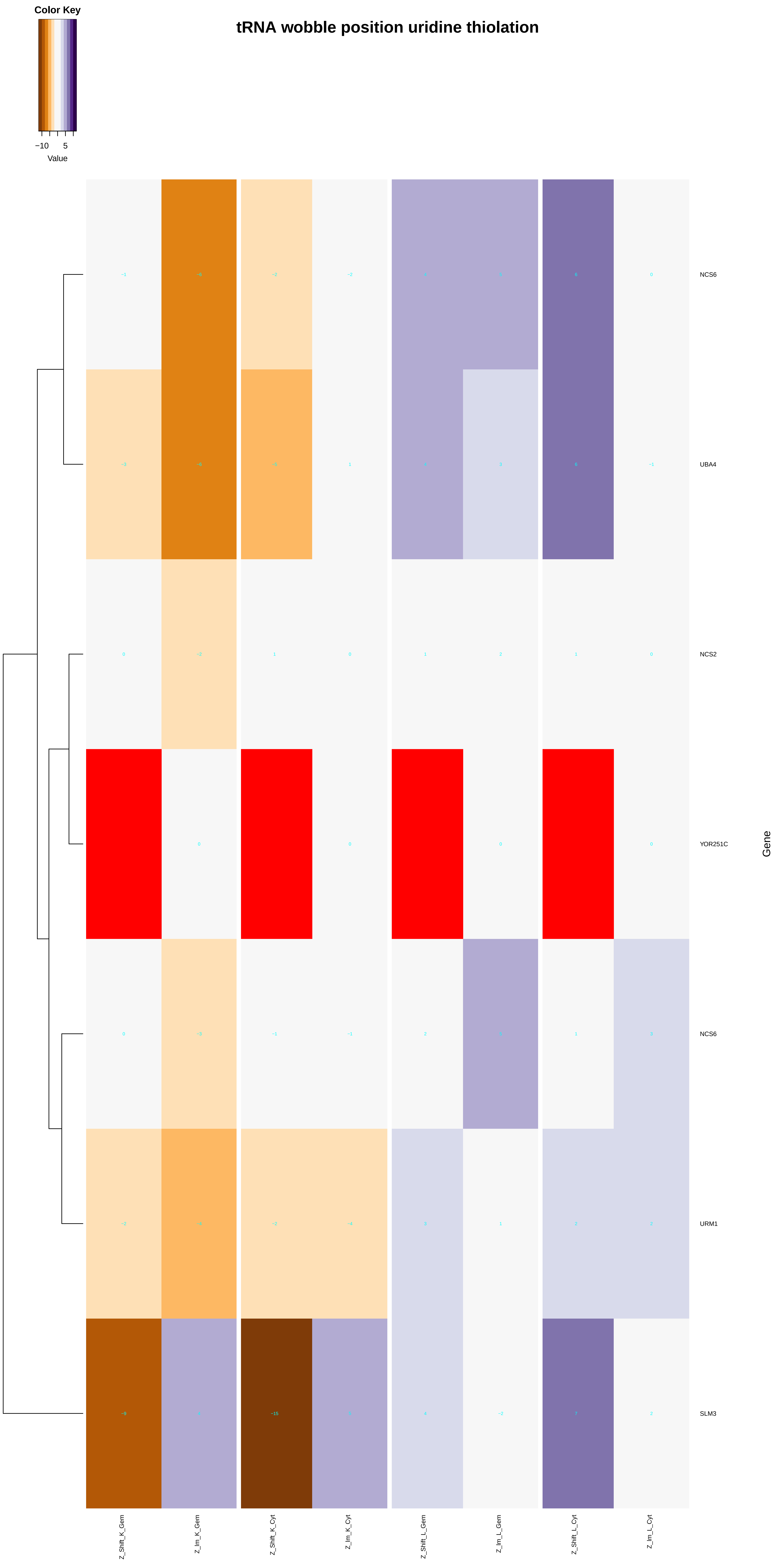
Color Key
−10 5
Value
tRNA wobble position uridine thiolation
NCS6
UBA4
NCS2
YOR251C
NCS6
URM1
SLM3
Gene
Z_Shift_K_Gem
Z_Im_K_Gem
Z_Shift_K_Cyt
Z_Im_K_Cyt
Z_Shift_L_Gem
Z_Im_L_Gem
Z_Shift_L_Cyt
Z_Im_L_Cyt

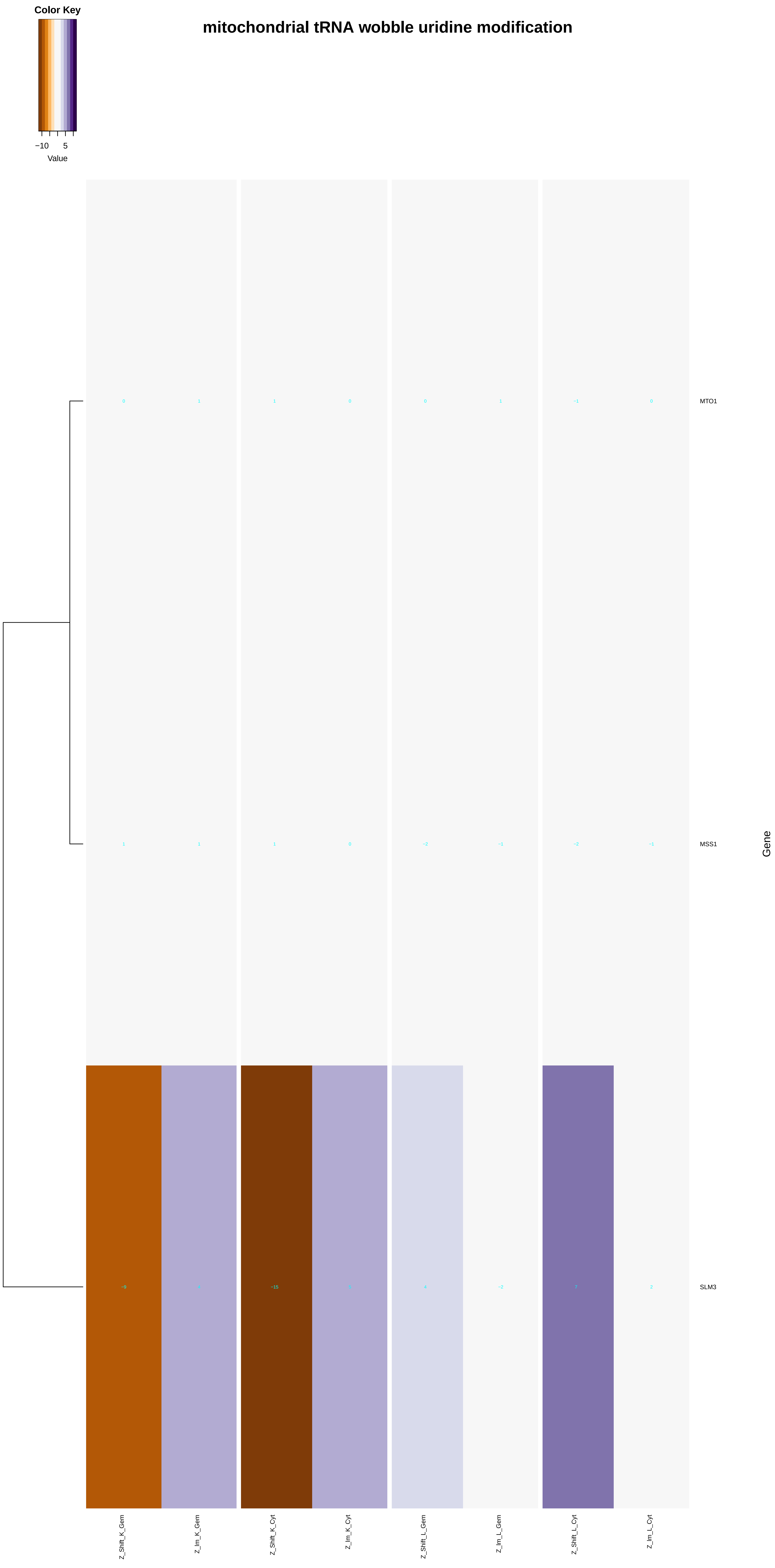

Color Key
−10
5
Value
mitochondrial tRNA wobble uridine modification
MTO1
MSS1
SLM3
Gene
0
1
1
0
0
1
−1
0
1
1
1
0
−2
−1
−2
−1
−9
4
−15
5
4
−2
7
2
Z_Shift_K_Gem
Z_Im_K_Gem
Z_Shift_K_Cyt
Z_Im_K_Cyt
Z_Shift_L_Gem
Z_Im_L_Gem
Z_Shift_L_Cyt
Z_Im_L_Cyt
